# Systemic application of bone-targeting peptidoglycan hydrolases as a novel treatment approach for staphylococcal bone infection

**DOI:** 10.1128/mbio.01830-23

**Published:** 2023-09-28

**Authors:** Anja P. Keller, Markus Huemer, Chun-Chi Chang, Srikanth Mairpady Shambat, Caroline Bjurnemark, Nicole Oberortner, Michaela V. Santschi, Léa V. Zinsli, Christian Röhrig, Anna M. Sobieraj, Yang Shen, Fritz Eichenseher, Annelies S. Zinkernagel, Martin J. Loessner, Mathias Schmelcher

**Affiliations:** 1 Institute of Food, Nutrition and Health, ETH Zurich, Zurich, Switzerland; 2 Department of Infectious Diseases and Hospital Epidemiology, University Hospital Zurich, University of Zurich, Zurich, Switzerland; Institut Pasteur, Paris, France

**Keywords:** antibiotic resistance, bacteriophages, phage display, cell-penetrating homing peptide, endolysin, MRSA, osteomyelitis, peptidoglycan hydrolase, protein therapeutics, *Staphylococcus aureus*, tissue-targeting

## Abstract

The current standard of treatment for chronic staphylococcal osteomyelitis entails high doses of antibiotics over the course of several weeks. Biofilm-associated and intracellular persisters are key factors contributing to therapeutic failure. Additionally, systemic application results in low concentrations of antibiotics at local infection sites due to its general distribution throughout the host. In this study, we explored a targeted approach for the treatment of staphylococcal osteomyelitis, employing a combination of highly active peptidoglycan hydrolases (PGHs) and cell-penetrating homing peptides (CPHPs) with specificity for osteoblasts. *In vitro* phage display on murine osteoblasts followed by next-generation sequencing led to the identification of 10 putative cell-penetrating homing peptides, which subsequently showed cell-line specific internalization of covalently linked fluorescent molecules into murine osteoblasts. Upon intravenous application, the lead candidate peptide mediated tissue-specific accumulation of an associated PGH in murine bones, confirming its function as an osteotropic peptide with cell-penetrating abilities. Furthermore, we selected three enzymes with high staphylolytic activity in murine serum screened from a set of 28 PGHs highly active against *Staphylococcus aureus* in human serum and under intracellular conditions: lysostaphin (LST), M23LST(L)_SH3b2638, and CHAPGH15_SH3bAle1. Finally, we demonstrated increased efficacy of the three PGHs modified with two osteotropic CPHPs as compared to their unmodified parentals at reducing bacterial numbers in a murine model of *S. aureus* deep wound subcutaneous infection leading to dissemination to the bone. Collectively, our findings show that modification of PGHs with tissue-specific CPHPs presents a viable approach for the systemic treatment of localized infections associated with intracellular bacteria.

**IMPORTANCE:**

The rising prevalence of antimicrobial resistance in *S. aureus* has rendered treatment of staphylococcal infections increasingly difficult, making the discovery of alternative treatment options a high priority. Peptidoglycan hydrolases, a diverse group of bacteriolytic enzymes, show high promise as such alternatives due to their rapid and specific lysis of bacterial cells, independent of antibiotic resistance profiles. However, using these enzymes for the systemic treatment of local infections, such as osteomyelitis foci, needs improvement, as the therapeutic distributes throughout the whole host, resulting in low concentrations at the actual infection site. In addition, the occurrence of intracellularly persisting bacteria can lead to relapsing infections. Here, we describe an approach using tissue-targeting to increase the local concentration of therapeutic enzymes in the infected bone. The enzymes were modified with a short targeting moiety that mediated accumulation of the therapeutic in osteoblasts and additionally enables targeting of intracellularly surviving bacteria.

## INTRODUCTION

Infections of the bone pose a severe health threat, as globally increasing incidence rates of osteomyelitis have been reported over the last few decades (1). *Staphylococcus aureus* is the major causative agent linked to the disease, being responsible for up to 60% of cases (1, 2). The current treatment regime for chronic osteomyelitis involves high doses of antibiotics over the course of several weeks, and surgical debridement of infected sites (3). Despite these efforts, a recurrence rate of 30% within the first year has been reported for adults diagnosed with chronic osteomyelitis (4). Prolonged treatment duration or treatment failure is a result of the ability of *S. aureus* to form biofilms and invade host cells, where they can persist, shielded from the host immune system and non-cell-permeable antibiotics (5 – 9). The capability of *S. aureus* to invade osteoblasts has been demonstrated in several *in vitro* studies and in a recent clinical case study on a patient with chronic osteomyelitis (10 – 12). Furthermore, the emergence of multi-drug resistant *S. aureus* has rendered the treatment of staphylococcal infections increasingly difficult. Resistance against glycopeptide and lipopeptide antibiotics including vancomycin and daptomycin, which are considered last resort therapeutics for the treatment of methicillin-resistant *S. aureus* (MRSA), has become gradually more prevalent over the years (13 – 17). Simultaneously, the development of novel antimicrobials has been steadily decreasing since the 1980s, creating a need for new therapeutic approaches and antimicrobials with novel mechanisms of action (18).

One potential class of new antimicrobials is peptidoglycan hydrolases (PGHs), enzymes that cleave specific bonds within the bacterial peptidoglycan and thereby cause cell lysis. These enzymes are especially suitable against Gram-positive bacteria, such as *S. aureus*, where the peptidoglycan is exposed to the environment due to the lack of an outer membrane (19). PGHs are derived from a variety of sources, including bacteria (bacteriocins), mammals (lysozymes), and bacteriophages (endolysins) (19 – 23). Two examples of bacteriocins targeting *S. aureus* are LST and ALE-1, which are produced by *Staphylococcus simulans* and *Staphylococcus capitis*, respectively (24, 25). The two homologous enzymes possess an N-terminal catalytic domain conferring glycylglycine endopeptidase activity, and a C-terminal domain of the SH3b cell wall-binding family (25 – 28). Endolysins from bacteriophages targeting Gram-positive bacteria display a similar modular architecture composed of enzymatically active domains and cell wall-binding domains (CBD), which confer catalytic activity and target specificity, respectively (19, 29). This modularity facilitates the engineering of chimeric enzymes by domain swapping, allowing specific optimization for an application of interest (19, 30, 31). Due to the high CBD-mediated specificity on a genus, species, and even strain level, off-target effects and bystander selection on beneficial organisms of the microbiome are reduced considerably (32). Unlike many commonly used antibiotics, the activity of PGHs is not dependent on active cell division, rendering them effective against metabolically inactive persister cells (33). Furthermore, the occurrence of resistance against endolysins is deemed improbable, as they target highly conserved moieties in the bacterial cell wall as a consequence of the viral-bacterial arms race (30, 34). The therapeutic potential of parental and chimeric PGHs has been demonstrated in several *in vitro* and *in vivo* studies, including animal models of bacteremia, skin infections, mastitis, endocarditis, osteomyelitis, and pneumonia (35 – 45).

Despite their therapeutic potential, some challenges remain in the systemic application of PGHs to treat localized staphylococcal infections, such as osteomyelitis. These include rapid clearance from the bloodstream, weak cell-penetrating properties, and insufficient accumulation at local infection sites (22, 46, 47). Several approaches to target intracellular bacteria have been proposed, including the administration of high doses of cell-permeable antibiotics (48), encapsulation of the antibiotic in nanoparticles or liposomes, and the modification of PGHs with cell-penetrating peptides (40, 44, 49, 50). Despite their ability to improve therapeutic efficacy against intracellular targets, cell-penetrating peptides do not confer tissue-specificity and, therefore, could accumulate in any cell type encountered upon systemic administration. Thus, the likelihood that they accumulate at an infection site at concentrations high enough to clear the infection is rather low.

Surfaces of tissues, organs, and cells are characterized by the expression of specific molecules. This heterogeneity can be utilized in active drug targeting via the modification and functionalization of the therapeutic with targeting moieties, such as homing peptides (HPs). HPs recognize and bind tissue-specific markers and can additionally confer intracellular uptake of an associated cargo by receptor-mediated endocytosis (51, 52). HPs with internalization properties are referred to as cell-penetrating homing peptides (CPHPs) and are of specific interest in applications with an intracellular target. Phage display is an established method for the identification of targeting peptides and has led to the discovery of peptides with specificity for a variety of organs and cell types, including the lung, intestine, skeletal and cardiac muscle, brain, and heart (53 – 56). Three similar homing peptides that showed specificity to bone resorption sites (D-Asp8) (57), bone formation sites (Asp-Ser-Ser)_6_ (58), and osteoblasts (SDSSD) (59) upon systemic application in mice have recently been published. In addition, Sun and colleagues have identified the receptor of SDSSD to be periostin (OSF-2) and demonstrated the targeted delivery of encapsulated siRNAs to bone resorption surfaces *in vivo* (59).

Here, we explore a targeted approach for the treatment of staphylococcal bone infection, employing a combination of highly active PGHs and CPHPs with specificity for osteoblasts. We screened a library of 28 PGHs that had previously been shown to be highly active in human serum and intracellular conditions (44, 45) to select PGHs with high staphylolytic potential in murine serum. Furthermore, we utilized a cell culture-based phage display approach and next-generation sequencing (NGS) to identify putative CPHPs with specificity for murine osteoblasts. The ability of 10 CPHP candidates to mediate cell line-specific internalization of a covalently linked fluorescent protein was assessed by fluorescence microscopy. To expand this concept, the biodistribution of a parental and CPHP-modified PGH was assessed in mice to demonstrate the bone-specific accumulation conferred by the CPHPs *in vivo*. Finally, the efficacies of three parental PGHs and their CPHP-modified counterparts were assessed in a murine model of deep wound subcutaneous infection leading to dissemination to the bone.

## RESULTS

### Identification of peptidoglycan hydrolases with high activity in murine serum for *in vivo* testing

**Fig 1:**
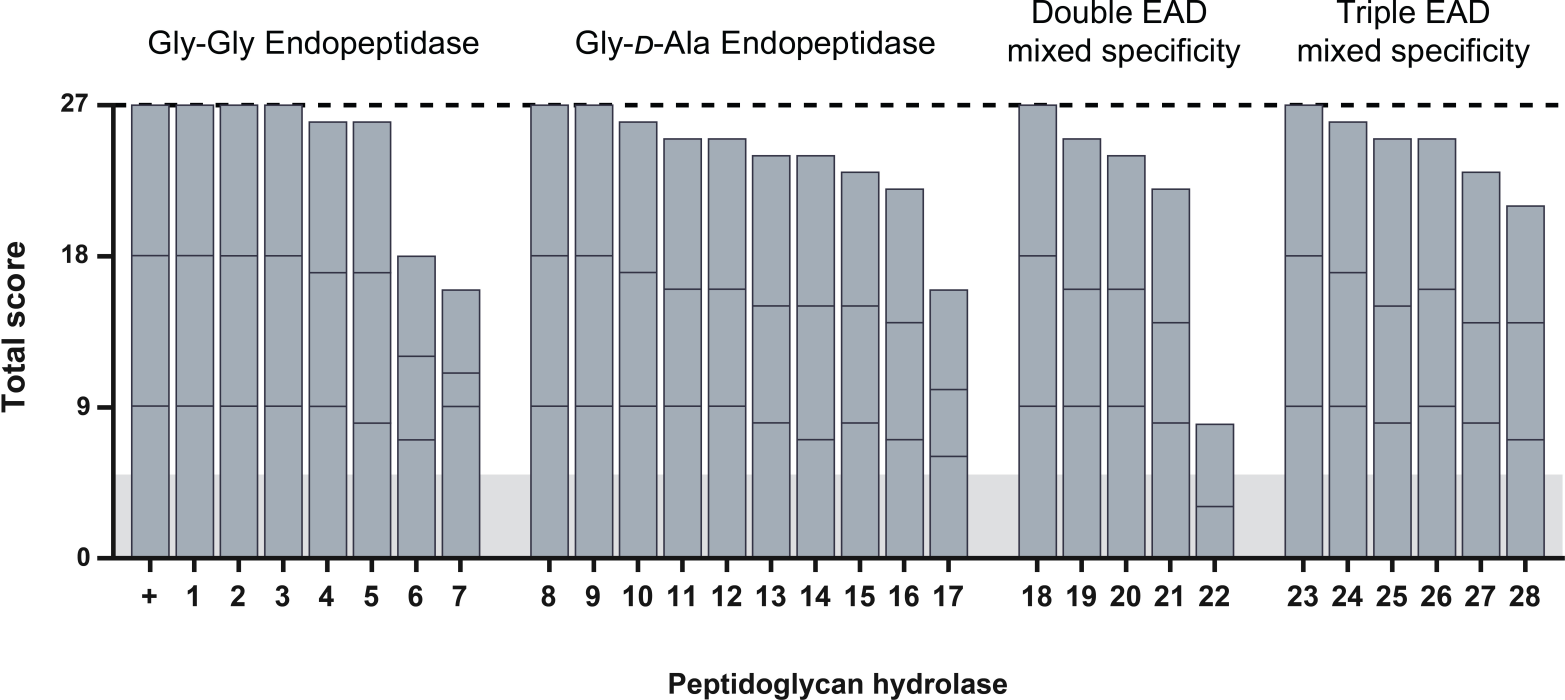
Screening of pre-selected PGHs for high staphylolytic activity in murine serum. Twenty-eight highly active enzymes were expressed in a microwell plate format and challenged with 10^7^ CFU/mL *S. aureus* Newman in murine serum for 30 minutes. Each enzyme was scored based on the number of surviving bacteria (>20 colonies = 0; 10–20 colonies = 1; <10 colonies = 2; and 0 colonies = 3). Experiments were performed in technical and biological triplicates, resulting in a maximum possible score of 27 (dashed line). Stacked bars indicate the scores reached in individual biological replicates. As controls, pET21a_LST (+) and pET21a (horizontal gray bar) were used. PGHs are sorted according to their enzymatic activity profile, the domain structure is detailed in Table S2.

**Fig 2:**
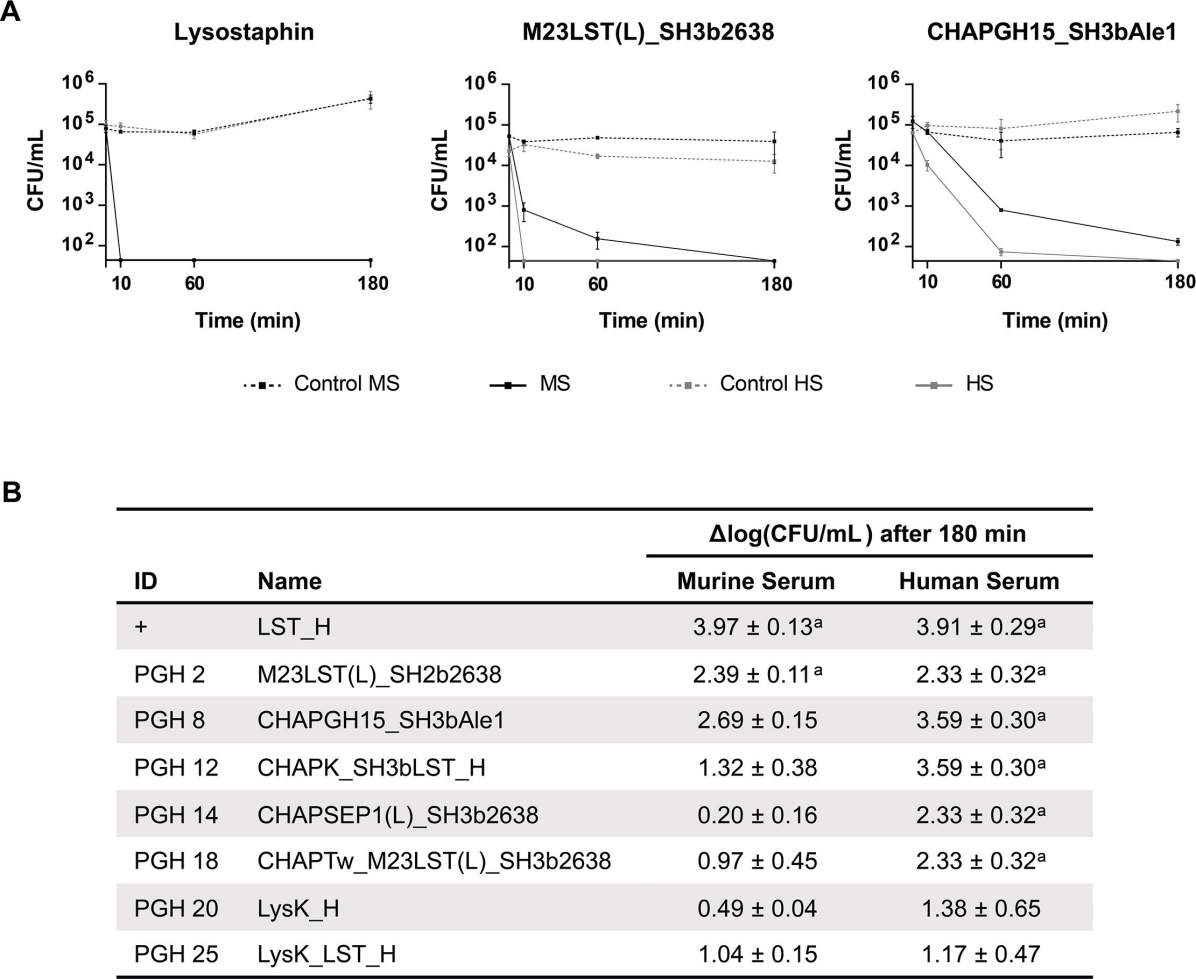
Comparison of *in vitro* staphylolytic activity of PGHs in murine and human sera. *S. aureus* Cowan I was treated with 20 nM of eight selected PGHs with high activity in murine and human sera. Bacterial counts were determined after 10, 60, and 180 minutes by plating. (**A**) Representative graphs for three PGHs show the mean (±SEM) CFU/mL determined at each timepoint in biological triplicates. *Y*-axis was cut at the detection limit (44 CFU/mL). (**B**) Table of average log(CFU/mL) reductions (±SEM) reached after 180 minutes compared to the untreated control for all tested enzymes. Averages were calculated from biological triplicates. Cases where the bacterial load was reduced to the detection limit are specified as ^a^.

Based on previous work (44, 45, 60), a selection of 28 PGH constructs with high activity and stability in human serum and intracellular environments (Table S2) were screened for activity in murine serum, taking into consideration downstream applications in a murine model of staphylococcal osteomyelitis. Under these conditions, 24 out of 28 constructs showed high staphylolytic potential similar to LST, which consistently reduced bacterial numbers to undetectable levels (Fig. 1). The high overall scores in the presented screening indicate that enzymes selected for activity in human serum in most cases also demonstrate high activity in murine serum. To gain further insight into this relation, eight high-scoring constructs were chosen, representing four mechanistic groups (Fig. 2B, Table S2). PGHs were expressed and purified, and their staphylolytic activity was assessed in time-kill assays in murine and human sera (Fig. 2). The small M23LST-containing constructs LST and M23LST(L)_SH3b2638 reduced the bacterial load to undetectable levels in both sera, confirming the results obtained in the initial screening. For the remaining constructs, the activity was generally lower in murine serum. The differences in activity in the two sera varied greatly between constructs, ranging from a mere 0.3-fold difference for CHAPGH15_SH3bAle1 to a 35.5-fold difference for CHAPSEP1(L)_SH3b2638. Based on these findings, LST, CHAPGH15_SH3bAle1, and M23LST(L)_SH3b2638 were chosen for further experiments since our *in vitro* data suggest that they might be promising candidates for systemic applications in both mice and humans.

### Selection of CPHP candidates with putative specificity for the bone niche by phage display and next-generation sequencing

**Fig 3:**
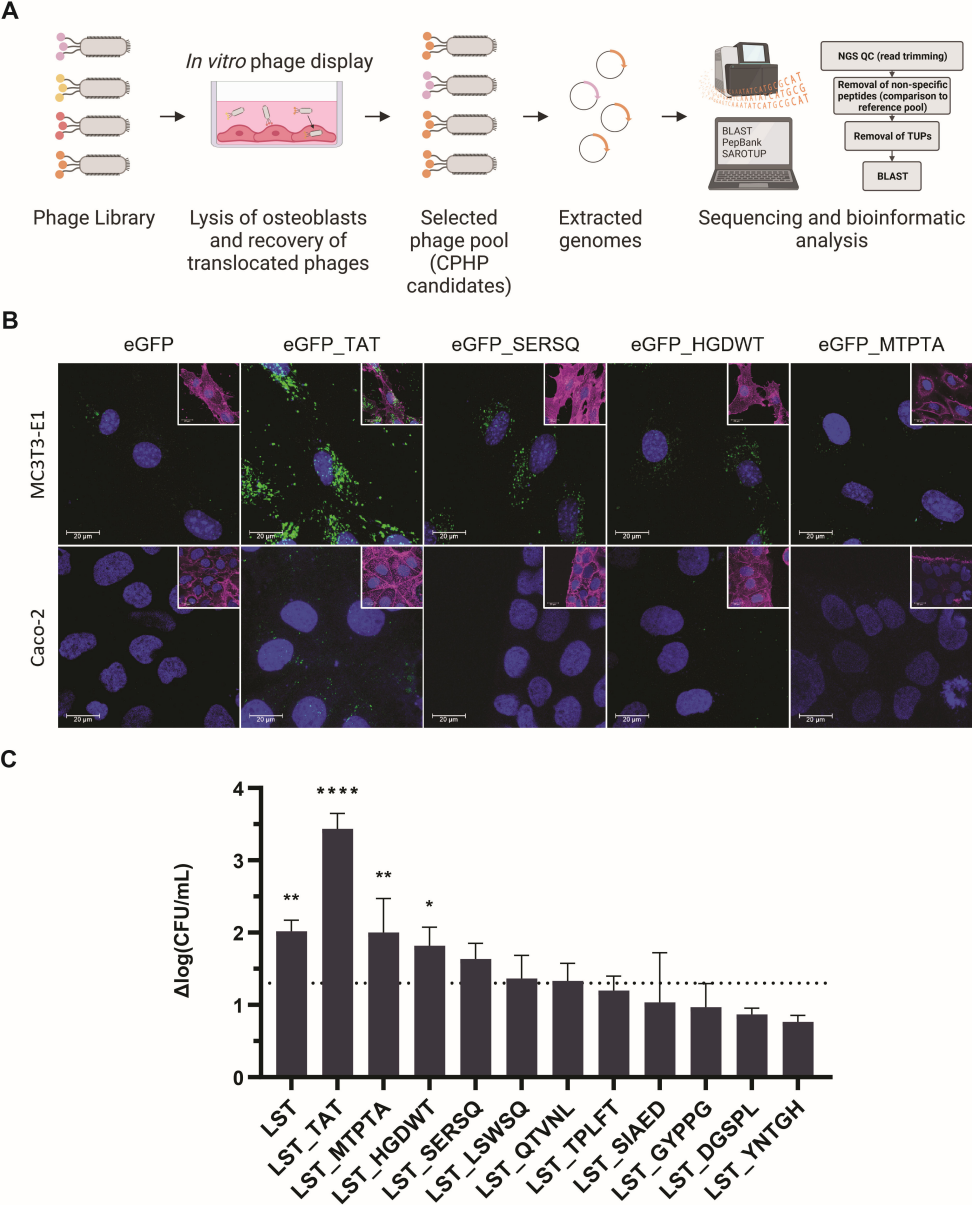
Selection and characterization of CPHP candidates. (**A**) CPHP candidates were identified using a cell culture-based phage display approach followed by next-generation sequencing and bioinformatics analysis. Ten CPHP candidates were C-terminally fused to eGFP (**B**) or LST (**C**) to assess their cell-line specificity and ability to translocate an active PGH cargo. (**B**) The cell line-specific uptake of eGFP and CPHP-modified eGFP was assessed by CLSM. MC3T3-E1 (top row) and a non-target cell line (Caco-2, bottom row) were treated for 60 minutes with 5 µM eGFP_CPHP or the controls eGFP and eGFP_TAT (a non-tissue-specific cell-penetrating peptide). Extracellular proteins were removed with three DPBS washes. Cells were stained with FM4-64 (membrane, magenta) and Hoechst 33342 (DNA, blue). Images for the three CPHP candidates that were subsequently used for *in vivo* experiments are shown. For ease of visualization, an overlay including the stained membrane is shown separately (inset) to the overlay of the eGFP and nucleic acid signals. (**C**) MC3T3-E1 cells were infected with *S. aureus* Cowan I (MOI = 5, 1 hour) and treated with LST, LST_TAT, or different LST_CPHP constructs (2 µM, 4 hours). Intracellular bacteria were enumerated by plating, and the average log(CFU/mL) reduction compared to an untreated control was determined (±SEM). The dashed line indicates a 95% reduction in intracellular CFUs. Experiments were conducted in biological triplicates. *P ≤ 0.05; **P ≤ 0.01; and ****P < 0.0001.

To identify bone-specific CPHP candidates, an *in vitro* phage display was established in a murine osteoblast cell line and adapted to select for phages that translocate into the cells (Fig. 3A). To gain deeper insight into the peptides present in the recovered phage pool, NGS was performed after one round of panning (61). NGS also markedly increases efficiency, circumventing the individual sequencing of hundreds of phage clones. From the selected phage pool, genomic regions encoding the 12-mer peptides with putative cell-penetrating homing characteristics were amplified by PCR. Amplicons were sequenced by Illumina sequencing, which returned 6,331,078 forward reads of 151 bp length. Reads were subjected to several processing steps: (i) trimming of sequences adjacent to the 36 bp inserts encoding the displayed peptides and eliminating any resulting sequences that were not of 36 bp length. The majority of discarded sequences originated from wild-type M13 phages without an insert. This resulted in 5,576,154 sequences encoding 79,732 unique peptides. The read count for each sequence was used as an approximation of abundance in the phage pool. (ii) Identification of peptides unique to the osteoblast pool by comparison to a peptide pool obtained from a panning on a non-target cell line (primary murine heart endothelial cells; Keller et al., unpublished data). The 11,198 peptides observed in both pools were eliminated based on a lack of specificity. (iii) Elimination of other target-unrelated peptides (TUPs). As a result, 21,931 peptides were eliminated, as they were predicted to be possible TUPs using the TUPredict tool, leaving only 51 peptides with a read count of over 400, which was set as a cut off for peptides with relatively high abundance in the selected pool. One underlying hypothesis of the applied phage display is that the selected peptides mimic the physiological interaction between a cell-surface receptor and a receptor-binding protein, possibly resulting in receptor-mediated endocytosis of the peptide and its associated cargo. Therefore, sequence similarity between the selected peptides and known proteins associated with osteoblasts and the bone extracellular matrix was used as a further selection criterion. The 51 peptides with the highest read count within the remaining selection were BLASTed against murine and human proteins available in the SwissProt/UniProt database. Based on this methodology, 10 peptides with putative cell-penetrating characteristics and specificity for murine osteoblasts were selected.

### CPHP candidates mediate osteoblast-specific uptake of an associated cargo molecule

To confirm target cell specificity and translocation capabilities of the 10 selected CPHP candidates in osteoblasts, peptides were C-terminally fused to the fluorescent protein eGFP. Cellular uptake of the labeled peptides was assessed by confocal laser scanning microscopy (CLSM) in the target cell line MC3T3-E1 (pre-osteoblasts) and a colorectal adenocarcinoma-derived non-target cell line (Fig. 3B; Fig. S1). Proteins were incubated for 1 hour with eukaryotic cells at equimolar concentrations. TAT, a general cell-penetrating peptide that internalizes in a plethora of eukaryotic cells, was used as a control (62). Localized intracellular eGFP signals were observed for all 10 candidates in osteoblasts, but not for Caco-2 cells, suggesting cell-line specific uptake of the CPHPs. In osteoblasts, differences in signal intensities were observed, representing a range of high (TAT, SERSQ, SIAED, YNTGH, GYPPG) and moderate (HGDWT, MTPTA, QTVNL, DGSPL, TPLFT, LSWSQ) uptake compared to the unmodified negative control eGFP. As this study had the dual aim of specifically targeting PGHs to local infections in osteoblasts and increasing the treatment efficacy by the eradication of intracellular *S. aureus*, we next set out to assess the intracellular activity of a CPHP-modified PGH in cultured MC3T3-E1 cells. To this end, the *in vitro* activities of the highly staphylolytic and well-described PGH LST, as well as TAT and CPHP fusions thereof were first determined in a cell culture medium to assess the potential detrimental effects of the attached peptides on the activity of the enzyme (44, 45, 63). The mean log reductions in *S. aureus* numbers obtained for all peptide-tagged LST variants were approximately 0.5 to 1.0 log units below the one obtained for the parental LST, although these differences were not statistically significant (Fig. S2). Equimolar amounts of modified and parental LST were then used to treat osteoblasts infected with *S. aureus* Cowan I. Treatment with the modified enzymes LST_TAT (*P* < 0.0001), LST_MTPTA (*P* = 0.042), and LST_HGDWT (*P* = 0.0095) resulted in a significant reduction in intracellular bacterial levels compared to an untreated control (Fig. 3C). These findings provide further evidence for the cell line-specific cell-penetrating characteristics of CPHPs identified by phage display, and their ability to internalize an associated proteinaceous cargo *in vitro*.

### HGDWT and SDSSD mediate accumulation of a PGH-cargo in murine bone tissue after systemic application

**Fig 4:**
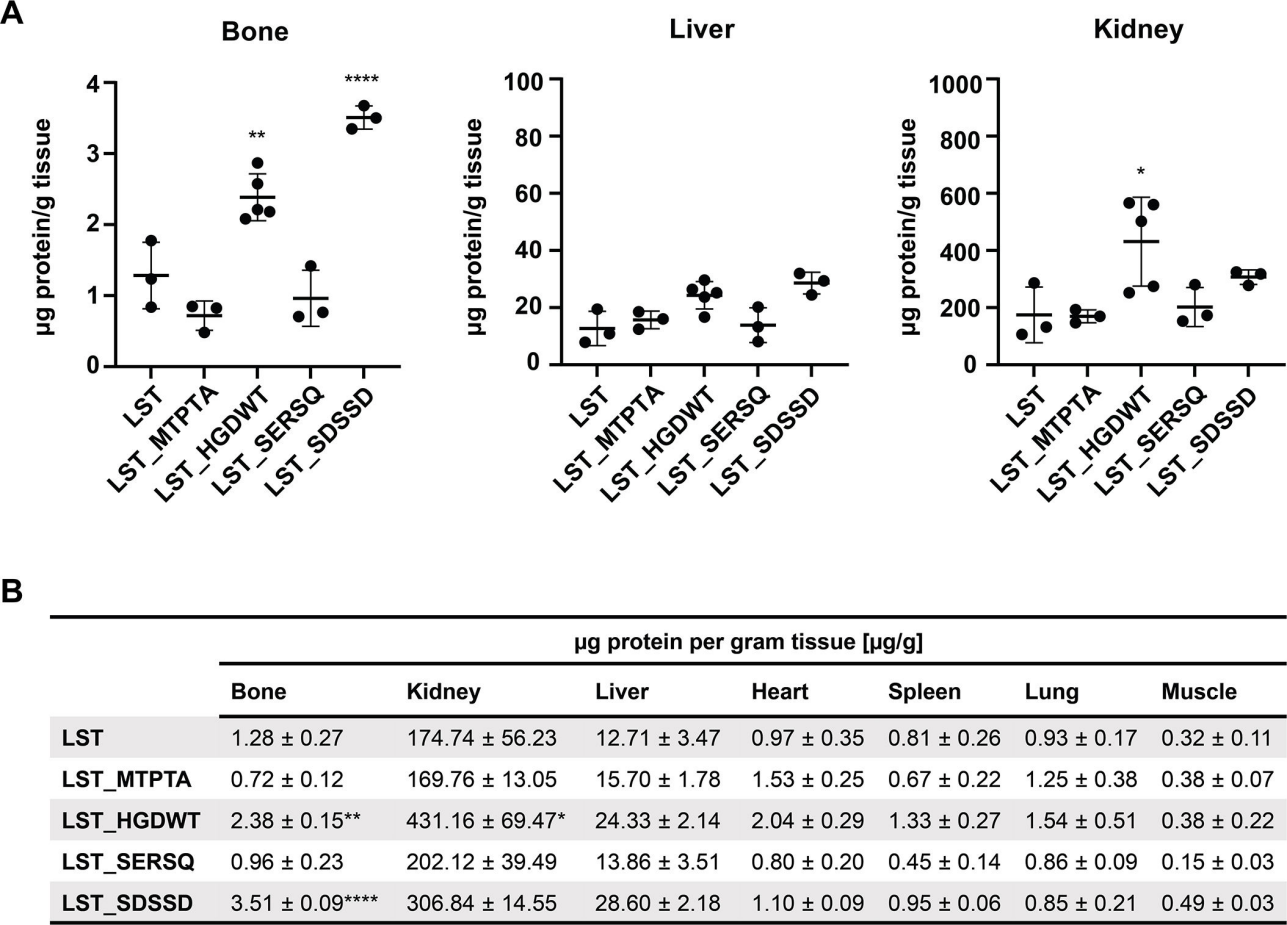
Biodistribution of a parental PGH and CPHP-modified variants thereof in murine tissues. Europium-labeled LST and LST_CPHPs were injected in C57BL/6 mice through the tail vein (10 mg/kg of body weight). Tissues were harvested 12 hours post-injection. Protein levels were determined by measuring europium signals in homogenized tissues by time-resolved fluorescence (TRF) spectrophotometry. Measurements were fitted to a standard curve determined for each tissue individually. (**A**) The amount of protein per mass of tissue (±SD) for bone, liver, and kidney is shown. Each individual data point represents one animal. Asterisks represent significant differences to the LST control. *P ≤ 0.05; **P ≤ 0.01; and ****P < 0.0001. (**B**) Summary of the numerical data obtained for all tested tissues (± SEM).

After having demonstrated their osteoblast-specific homing properties *in vitro*, the tissue-specific accumulation of the three best-performing peptides (MTPTA, HGDWT, and SERSQ), as well as SDSSD (59) as a reference, was determined *in vivo.* Biodistribution in mice was determined for CPHPs C-terminally fused to LST as a representative PGH cargo and labeled with DTBTA-Eu^3+^ to enable detection via time-resolved fluorescence measurements. The mass spectra obtained for ATBTA-Eu^3+^ and DTBTA-Eu^3+^ are shown in Fig. S3. Labeled proteins were intravenously injected into mice at a concentration of 10 mg protein per kg of body weight. At 12 hours post-injection, tissues of interest were extracted and homogenized, and europium signals were measured in each tissue. Signal intensities were compared to standard curves to quantify the amount of protein in each tissue (Fig. 4). Significantly higher levels of LST_HGDWT (*P* = 0.033) and LST_SDSSD (*P* = 0.009) as compared to the parental LST were observed in bone, but not in other tissues (with the exception of HGDWT in kidneys), whereas LST_SERSQ and LST_MTPTA did not show the same tissue-specific accumulation. These results confirm the ability of the CPHPs HGDWT and SDSSD to increase the local concentration of PGH cargo in the targeted tissue after systemic application.

### CPHP-fused PGHs exhibit enhanced efficacy in a murine deep wound subcutaneous infection model

**Fig 5:**
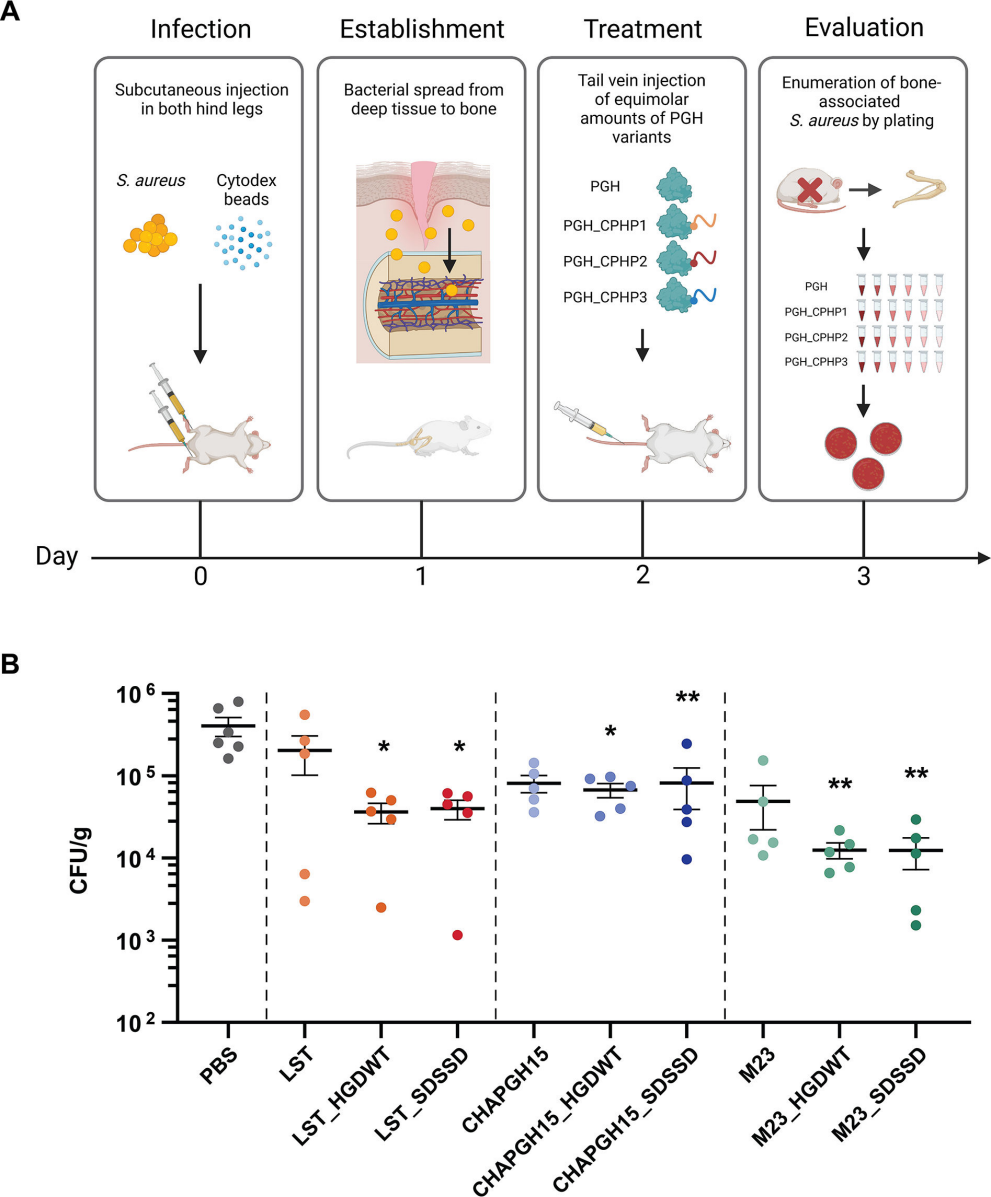
Treatment efficacy of parental and CPHP-modified PGHs in a murine deep wound subcutaneous infection model. (**A**) Experimental design of the *in vivo* efficacy study. Hind legs of mice were infected subcutaneously with *S. aureus* Cowan I, and infection of the bone was established over the course of 48 hours. Mice were treated with a single dose of parental PGH or PGH_CPHP (150 µL, 75 µM) or PBS as a control. At 14–16 hours post-treatment, the femur, fibula, and tibia were harvested, homogenized, and total CFUs normalized to the weight of the bone were determined by plating. (**B**) *S. aureus* CFUs (±SEM) in murine bones after treatment with parental PGHs and their CPHP-modified variants as compared to a PBS control. *P ≤ 0.05 and **P ≤ 0.01.

Finally, to assess the efficacy of CPHP-modified PGHs in a murine deep wound subcutaneous infection model leading to dissemination to the bone, HGDWT and SDSSD were fused to the PGHs LST, CHAPGH15_SH3bAle1 (CHAPGH15), and M23LST(L)_SH3b2638 (M23). The cytotoxicity of endotoxin-free CPHP-fused variants and parental enzymes was determined *in vitro* in murine osteoblasts, and less than 5% cytotoxicity was observed for all constructs (Fig. S4). Prior to the *in vivo* experiments, the staphylolytic activity of all enzymes was confirmed in a time-kill assay in murine serum (Fig. S5). All CPHP variants showed comparable activity to their parental PGH, causing a >2 log unit reduction in bacterial numbers, with the exception of M23_HGDWT, which reduced the bacterial load by only 1.72 (±0.06) log units. For the *in vivo* study, mice were infected subcutaneously with *S. aureus* Cowan I in both hind legs and infection of the bone was established over the course of 48 hours. Mice were treated by tail vein injection of a single dose of PGH_CPHP or parental PGH (Fig. 5A). At 14–16 hours post-treatment, the femur, fibula, and tibia of both hind legs were harvested and homogenized, and the number of surviving bacteria per relative bone mass was determined. Phosphate-buffered saline (PBS)-treated control mice showed average infection levels of 4.0 × 10^5^ (±1.0 × 10^5^) CFUs. While the differences in *S. aureus* reduction between animals treated with PGH_CPHP constructs and those treated with the respective parental enzymes were not statistically significant, it was found that only the CPHP-modified PGHs, but not unmodified parentals (*P* > 0.05), have significantly reduced levels of bone-associated *S. aureus* as compared to the PBS control (LST_HGDWT, *P* = 0.020; LST_SDSSD, *P* = 0.024; CHAP_HGDWT, *P* = 0.024; CHAP_SDSSD, *P* = 0.009; M23_HGDWT, *P* = 0.006; and M23_SDSSD, *P* = 0.005) (Fig. 5). These findings confirm the observations made in the biodistribution experiment and highlight the beneficial effect of the addition of cell-penetrating homing peptides to systemically administered peptidoglycan hydrolases to treat localized staphylococcal infections.

## DISCUSSION

The current standard of treatment for chronic staphylococcal osteomyelitis entails high doses of intravenously administered antibiotics over the course of several weeks and debridement of infected sites (3, 64). Despite these invasive treatments, a recurrence rate of 30% within the first year has been reported for adults diagnosed with chronic osteomyelitis (4). Intracellular persisters and the widespread occurrence of antibiotic resistance are the key factors contributing to recurrent infections and treatment failures (65). Furthermore, systemically administered therapeutics are often present in insufficient concentrations at localized infection sites due to their general distribution throughout the organism. In this proof-of-concept study, we address the challenges associated with the treatment of staphylococcal bone infections. In a recent attempt to harness PGHs for treating such infections, Sumrall et al. (66) reported the inefficient translocation of PGHs to the infected bone in a mouse model of fracture-related infection. The locally administered PGHs reduced the infection in the surrounding soft tissue but did not affect bacterial numbers in the bone itself. Here, we demonstrate, for the first time, the amplified efficacy of a targeted treatment approach employing PGHs modified with cell-penetrating homing peptides.

We have identified a novel cell-penetrating homing peptide—HGDWTKRWSFLA—and demonstrated its ability to significantly increase the local concentration of an associated PGH in bone after intravenous administration in mice. This effect translated to its efficacy in a murine osteomyelitis model, where LST_HGDWT significantly reduced the bacterial load in infected bones by 1.2 log units, a 50% increase compared to the unmodified LST (0.8 log units). This relative increase in efficacy was also observed for the PGH M23LST(L)_SH3b2638 (1.5 vs 1.1 log units), and when the two enzymes were coupled to a different CPHP, SDSSD. The peptide had been identified by Sun et al. (59), who demonstrated the localized delivery of SDSSD-modified nanoparticles to bone formation surfaces after systemic injection in mice. We have shown that the *in vivo* homing characteristics of SDSSD were translatable to a proteinaceous cargo as demonstrated in biodistribution and efficacy studies presented here.

Tissue-specific peptides have been extensively studied in the context of targeted drug delivery, and phage display has prevailed as the method of choice for their identification (51, 52, 67 – 70). Here, we have performed *in vitro* phage display, selecting peptides with the ability to bind and internalize into murine osteoblasts. The classic phage display approach of repeated exposure to the target alternating with amplification of the selected phage pool in a bacterial host suffers from a strong amplification bias, which can favor phages encoding parasitic peptides over those selected in the panning step (71 – 74). As a consequence, we have drastically condensed the procedure, performing only one round of panning with subsequent NGS of the phage pool, as proposed by t’Hoen and colleagues (61). This approach proved successful, as 10 putative CPHPs mediated uptake of a covalently linked cargo molecule in osteoblasts, but not a non-target cell line, exhibiting the specific cell-penetrating capabilities we have selected for (Fig. 3B; Fig. S1). This CPHP-mediated cell penetration did not directly translate to an increased intracellular killing activity in a cell culture setting. Equimolar amounts of LST-CPHP constructs showed equal or reduced intracellular activity compared to unmodified LST in infected osteoblasts (Fig. 3C). This can be partially explained by the observation that the *in vitro* activity of LST was affected by the C-terminal modification (Fig. S2). This trade-off between PGH activity and added functionality (in this case, internalization) has been observed previously in a variety of studies (40, 44, 45, 47). This affects each combination of PGH and CPHP differently, as observed for the pairings assessed in the mouse model (Fig. S5). Despite the marked reduction in the *in vitro* activity of M23_HGDWT as compared to M23 at an equimolar concentration in murine serum, the construct showed significant activity in the deep wound subcutaneous infection model leading to dissemination to the bone, comparable to M23_SDSSD. It is possible that the amount of M23_HGDWT that accumulated in the bone resulted in a high enough local concentration to compensate for the difference in activity observed *in vitro*. This is corroborated by the *in vitro* data, where M23_HGDWT showed similar activity to M23_SDSSD if a higher concentration was applied (500 nM vs 20 nM; Fig. S5).

One parameter that could impact the ability of a PGH-CPHP construct to sufficiently accumulate at the target site within the body is the serum circulation half-life of the protein, which determines the time it remains in the circulation system. The half-lives in mice of all three parental PGHs investigated here in our murine infection model (i.e., LST, CHAPGH15, and M23) have previously been determined in our lab (45) and were found to be in a very similar range, with no significant differences between the proteins (alpha phase half-lives were all around 2 hours, and beta phase half-lives ranged from 32 to 52 hours). This being said, there is no reason to assume that adding CPHPs, i.e., short 12-amino acid peptides, to any of these proteins (whose molecular weights are in the range of 25–30 kDa) would result in significantly altered half-lives of these constructs via any of the well-described general clearance mechanisms such as renal filtration (which depends on the hydrodynamic volume of the protein), lysosomal degradation, or proteolytic cleavage (46, 75). One could speculate that the half-lives of the PGH-CPHP constructs may be longer than those of their parental PGH counterparts due to their increased bone-homing and -accumulation properties. However, this would be a specific and desired side effect of our tissue targeting strategy, likely contributing to the enhanced killing of *S. aureus* in bone tissue, which we observed with the CPHP-tagged variants. Overall, the results of our biodistribution study and the murine infection model suggest that the serum circulation half-lives in mice of all tested PGH-CPHP constructs are sufficient to allow for significant accumulation of the proteins in the targeted bone niche and profound reduction of *S. aureus* cells residing there. Another possible concern related to the half-lives of our constructs is that the measurement of Eu^3+^-labeled proteins accumulated in the target tissues in our biodistribution experiment could be confounded by proteins (or fluorescent fragments thereof) left in the circulation system at the time of organ harvesting and TRF measurement. However, the alpha phase half-life of LST, which is the protein we have used for this experiment, has been reported to be 1.9 hours (also determined via TRF measurement of Eu^3+^-labeled LST) (45), meaning that, after 12 hours, only approximately 1.5% of the initially injected LST concentration would be left in the circulation system. Therefore, the impact of residual protein or protein fragments in blood circulation on the measurement of LST or LST-CPHP internalized in cells of various tissues 12 hours after injection in our biodistribution study is expected to be rather minimal. The possibility that a fraction of the internalized proteins are degraded (with fluorescent fragments contributing to the total measured fluorescence signal) cannot be excluded. However, the fact that the results of our biodistribution study (i.e., increased concentrations of LST_HGDWT and LST_SDSSD in bone tissue as compared to LST; see Fig. 4) are in agreement with the findings of the murine infection model (i.e., significantly reduced *S. aureus* numbers in bone tissue after treatment with LST_HGDWT and LST_SDSSD, as opposed to LST; see Fig. 5) suggests that a considerable fraction of the internalized proteins must be present in intact and active forms at 12–16 hours post-injection.

The observation that LST and LST-CPHP concentrations measured in our biodistribution study largely differed between different tissues is not surprising. The liver and particularly kidneys are involved in the clearance of the proteins from the circulation system (46, 75), which means that proteins accumulate in these organs during the elimination process. Therefore, the substantially higher protein concentrations measured in kidney and liver tissues as compared to other tested tissues were to be expected. Furthermore, it was interesting to see that there were no significant within-tissue differences in any of the control (i.e., non-bone) tissues, with the exception of LST-HGDWT in kidney (see Fig. 4B). This suggests that adding various CPHPs to LST did not impact the accumulation of the protein in a majority of the tested non-bone tissues, whereas the addition of HGDWT and SDSSD significantly increased LST concentrations in bone tissue. The finding that slightly elevated concentrations of LST-HGDWT as compared to LST were found in most tissues suggests that HGDWT may be less selective for bone tissue than SDSSD. However, these differences were statistically significant only for bone (*P* ≤ 0.01) and kidney (*P* ≤ 0.05), indicating that HGDWT still has a strong preference for bone tissue, and treatment with an HGDWT-tagged endolysin results in stronger accumulation of the protein in bone tissue than in other tissues. A possible explanation for this observation would be that HGDWT interacts with a cell surface receptor that is present on cells of multiple tissues but strongly overexpressed only on bone (and to a lesser extent on kidney) cells.

Besides protein engineering for added functionality, the widely differing conditions encountered in therapeutic settings can be highly impactful on the activity of PGHs. Thus, previous studies have employed an iterative screening and characterization method to identify parental and chimeric PGHs with optimal staphylolytic activity in a specific environment (44, 45, 60). Here, we have expanded on these screenings to select enzymes that (in addition to high activity and stability in human serum and intracellular compartments) show high activity in murine serum, considering their assessment in a murine model in this study. The selected constructs represent a variety of enzymatic activities (endopeptidases, amidases, and combinations thereof), their domains originating from a diverse background of bacteriocins and bacteriophage-encoded endolysins. A vast majority of the screened enzymes retained high staphylolytic potential in murine serum, highlighting the effectiveness of the comprehensive selection and characterization procedure established in the preceding studies (Fig. 1). Eight constructs of interest were further compared quantitatively, as the initial screening approach may suffer from certain inherent biases since constructs are expressed from different vectors and host bacteria, which possibly affects their expression levels (60).

Time-kill assays revealed significant differences in activity, which were not evident from the data obtained in the screening (Fig. 2). Interestingly, most tested enzymes performed better in human serum than murine serum, independent of their structural and enzymatic composition. This was not confirmed for LST and M23LST(L)_SH3b2638, as they reduced bacterial numbers to the detection limit in both sera. A recent study has demonstrated the high variability in the activity of an endolysin in blood and serum sampled from a variety of model hosts (76). Notably, the authors observed that the activity of the endolysin was higher in the presence of human serum (as well as rabbit, dog, and horse) than the serum of mice (and rats), as a result of its synergistic interaction with human serum albumin and lysozyme. To this day, very few studies are available on the application of endolysins in animal models of staphylococcal osteomyelitis (39, 42). Recently, Karau et al. intravenously injected a single dose of the endolysin CF-301 in an *S. aureus* osteomyelitis model in rats 1-week post-infection (42). Although they reported a significant reduction in bacterial numbers localized in the bone compared to an untreated control group, the effect was relatively weak (<0.5 log units at ~15 mg/animal). In our animal model, the final three PGHs selected using the above-described approach showed higher efficacy even without the addition of a CPHP (up to 1.1 log reduction at ~0.2 mg/animal). Together, these findings highlight the necessity to specifically select enzymes optimally suited for the model organism of choice, in order to achieve optimal treatment efficacy.

In conclusion, our work provides proof-of-concept for the targeted delivery of systemically administered PGHs via fusion to CPHPs as a means of effectively reducing *S. aureus* numbers in infected bones. This represents another important step toward the applicability of PGHs as therapeutics against difficult-to-treat bacterial infections. Clearly, the further pre-clinical development program of potential new PGH-CPHP-based drugs would need to include studies conducted over longer periods of time, including repeated administration of the proteins, to evaluate their potential to clear such infections. Besides osteomyelitis, *S. aureus* is a major causative agent of a variety of other localized infections, including infective endocarditis, skin and soft tissue infections, and pleuropulmonary and device-related infections (1). We believe that the targeted approach described in this study may be applied to these diseases as well, as peptides homing to the vasculature of several tissues have been identified, including the lung, intestine, skeletal and cardiac muscle, brain, and heart (53 – 56).

## MATERIALS AND METHODS

### Bacterial strains and growth conditions

Bacterial strains used in this study are listed in Table S1. All bacteria were cultured at 37°C, shaking at 220 rpm. *S. aureus* were grown in tryptic soy broth (TSB). *Escherichia coli* ClearColi BL21(DE3) (Lucigen, Middleton, USA) for endotoxin-free protein expression were grown in an LB medium optimized for endotoxin-free protein expression (LB-PES) (47). All other *E. coli* were grown in LB medium, or LB-PE optimized for protein expression (47). *E. coli* cultures were supplemented with appropriate antibiotics (Sigma-Aldrich, St. Louis, USA), as detailed in Tables S1 and S2.

### Screening of PGH constructs for high staphylolytic activity in murine serum

PGH constructs were screened for high activity in murine serum in an established microwell plate setting (60). Previous work using the same screening platform had yielded enzymes featuring high staphylolytic activity in a variety of conditions relevant to different therapeutic settings (44, 45, 60). Based on this previous work, 28 constructs featuring high activity and stability in human serum and intracellular environments were chosen for further screening using mouse serum. These pre-selected constructs exhibit a broad range of enzymatic activity and can be mechanistically grouped into Gly-Gly endopeptidases (PGHs 1–7), Gly-d-Ala endopeptidases (PGHs 8–17), and enzymes with multiple enzymatically active domains (PGHs 18–28) (Table S2). The latter constructs include domains belonging to the two previous groups, or exhibiting additional aminohydrolytic activity (PGHs 19, 21–24, 27, 28). The domains represented in this selection originate from various PGHs, including the two bacteriocins LST (24) and ALE1 (25), and the endolysins of bacteriophages 2638A (77), K (78), GH15 (38), Twort (79), H5 (80), phi11 (81), λS2 (82), and SEP1 (83). For the screening in murine serum, *E. coli* strains listed in Table S2 were inoculated in triplicates on a 96-well plate and proteins were expressed as described (60). After the release of the expressed enzymes by chloroform-mediated cell lysis, mouse serum (preservative-free; Bio-Rad Antibodies, Hercules, USA) spiked with 10^7^ CFU/mL *S. aureus* Newman was added to each well, and the plate was incubated at 37°C for 30 minutes. Contents of the wells were diluted 10-fold in Dulbecco's phosphate-buffered saline(DPBS) (Thermo Fisher Scientific, Waltham, USA), spotted on LB agar plates, and incubated at 37°C overnight. As negative and positive controls, strains carrying an empty expression vector (pET21a) or expressing a highly active peptidoglycan hydrolase (pET21a_LST) were chosen, respectively. Each technical replicate was rated according to the following scheme: >20 colonies = 0; 10–20 colonies = 1; <10 colonies = 2; and 0 colonies = 3. Scores from technical and biological triplicates were added, resulting in a maximum possible score of 27.

### Expression and purification of proteins for *in vitro* application

Constructs for *in vitro* application were expressed essentially as described before (60). Briefly, *E. coli* strains harboring constructs of interest (Tables S2 and S3) were grown to mid-log phase in LB-PE with appropriate antibiotics, induced with 0.5 mM isopropyl ß-D-1-thiogalactopyranoside (IPTG) (Carl Roth GmbH, Karlsruhe, Germany) and incubated for 18 hours at 19°C, shaking at 150 rpm. Cells were harvested by centrifugation (7,000 × *g*, 10 minutes, 4°C) and subjected to one freeze-thaw cycle at −20°C. Pellets were resuspended in IMAC lysis buffer (300 mM NaCl, 50 mM Na_2_HPO_4_, 10 mM imidazole, and 30% glycerol, pH = 8) or cation exchange chromatography (CIEX) lysis buffer (50 mM Na_2_HPO_4_, 20% glycerol, pH = 7.4) and lysed by pressure homogenization (100 MPa; Stansted Pressure Cell Homogenizer; Homogenising Systems Ltd., Harlow, UK). Cellular debris was removed from the lysate by centrifugation (20,000 × *g*, 60 minutes, 4°C) and the supernatant was collected.

#### IMAC protein purification

His-tagged proteins were purified essentially as described previously (60) using a nickel-nitrilotriacetic acid agarose resin (ABT, Madrid, Spain). Briefly, the cleared lysate was mixed with the resin and incubated for 1 hour at 4°C under constant motion. Resin was washed with 30 column volumes of IMAC lysis buffer. Proteins were eluted with elution buffer (IMAC lysis buffer supplemented with 250 mM imidazole), and fractions with concentrations higher than 1 mg/mL were pooled.

#### CIEX protein purification

Untagged proteins were purified by CIEX on a 5 mL HiTrap SP FF column (Cytiva, Marlborough, USA) using an Äkta pure FPLC system (GE Healthcare, Chicago, USA), as described previously (44). Proteins were eluted in CIEX lysis buffer over an increasing NaCl gradient. Elution was monitored by UV detection at 280 nm and relevant peak fractions were pooled.

After either purification method, proteins were dialyzed overnight at 4°C against two changes of dialysis buffer (50 mM Na_2_HPO_4_, 300 mM NaCl, 30% glycerol, pH = 7.5) in a 6–8 kDa cutoff membrane (Spectra/Por Dialysis Tubing; Spectrum Lifesciences LLC, Rancho Dominguez, USA). Protein concentration was determined by spectrophotometric measurements (Nanodrop ND-1000; Thermo Fisher Scientific) and purity and size were verified by SDS-PAGE, using Criterion TGX Stain-Free precast gels (Bio-Rad) and subsequent Coomassie staining (InstantBlue; Abcam, Cambridge, UK).

### Endotoxin-free protein expression for *in vivo* applications

For the biodistribution and efficacy assessment of selected PGH and PGH_CPHP constructs in mice, proteins were expressed and purified in an endotoxin-free manner as described before (47). All vessels sterilized in-house used for buffer storage and large-scale culturing were decontaminated with a 0.5 M NaOH solution. Plasmids of the structure pET302_PGH_CPHP were transformed into ClearColi BL21(DE3) (Lucigen). Transformants were plated on LB-PES agar plates supplemented with 100 µg/mL ampicillin. ClearColi cultures carrying the expression vector of interest were grown to late-log phase, and expression and harvest of cell pellets were executed as described above. Cells were lysed by sonication (80% power, 5 × 1 minute, 1 second on, 1 second off; Sonopuls HD 2070.2; Bandelin, Berlin, Germany) and debris was removed by centrifugation (20,000 × *g*, 60 minutes, 4°C). The FPLC system and columns used for protein purification were thoroughly cleaned with 200 mL 0.5 M NaOH and 300 mL MQ, to remove residual endotoxins. CIEX was performed as described above. Enzymes used in biodistribution experiments were labeled with DTBTA-Eu^3+^ as described below. All endotoxin-free proteins were subjected to an additional purification step by size exclusion chromatography (SEC) to remove residual contaminations (host proteins, unbound DTBTA-Eu^3+^, cross-linked species). Proteins were purified on a HiLoad 16/600 Superdex 200 pg column (Cytiva) in SEC running buffer (50 mM Na_2_HPO_4_, 500 mM NaCl, 5% glycerol, pH = 7.4). Protein concentration was determined using the Pierce BCA Protein Assay Kit (Thermo Fisher Scientific) according to the instructions provided. Purity and size were verified by SDS-PAGE as described above. Endotoxin-free proteins were dialyzed into DPBS, and residual endotoxin levels were determined using the Endonext EndoZyme II Endotoxin Detection Assay Kit (bioMérieux, Marcy-L’Étoile, France). Protein batches with endotoxin levels less than 0.5 EU/mL were released for animal experiments. *In vitro* activity was assessed by time kill assays.

### Assessment of staphylolytic activity of PGHs

*In vitro* activity of PGHs was determined in time-kill assays essentially as previously described (60). In short, enzymes were diluted in serum or buffer of choice (10× the final concentration of 20 nM), and 20 µL each was transferred to a 96-well plate. An overnight culture of *S. aureus* was diluted in fresh TSB medium, grown to mid-log growth phase, and used to inoculate the desired medium to reach a concentration of 1.1 × 10^6^ CFU/mL. One hundred eighty microliters were added to the enzymes and incubated at 37°C. The inoculum and number of surviving bacteria after timepoints 10, 60, 120, or 180 minutes were determined from a dilution series and spot plating on LB agar plates. Plates were incubated overnight, and the number of colonies per milliliter was determined for each time point and plotted against the time.

### Cell culture

For general maintenance and all experimental incubation steps, eukaryotic cells were kept at 37°C in a 5% CO_2_ atmosphere. Murine pre-osteoblasts (MC3T3-E1 Subclone 4; ACC 210; DSMZ, Braunschweig, Germany) were maintained in Minimum Essential Medium alpha without ascorbic acid (AMEM; Thermo Fisher Scientific) supplemented with 10% fetal bovine serum (FBS) (Gibco, certified, OneShot; Thermo Fisher Scientific). Caco-2 cells (ECACC 86010202; Sigma-Aldrich) were cultured in DMEM (1×) + GlutaMax AX-I ([+] 4.5 g/L D-glucose, [+] 110 mg/L sodium pyruvate; Thermo Fisher Scientific) supplemented with 10% FBS and 1% MEM Non-Essential Amino Acids Solution (Thermo Fisher Scientific). Cells were grown in T75 flasks and split daily at a ratio of 1:2 or every other day at a ratio of 1:10.

### Selection of CPHPs by phage display on murine osteoblasts

Phage display is a well-established method for the selection of peptides with affinity for a target tissue, cell type, or molecule (67). An *in vitro* phage display for the selection of peptides with cell-line specific internalization characteristics (CPHPs) was carried out using the Ph.D.−12 Phage Display Peptide Library (NEB, Ipswich, USA) according to the recommendations by the provider and previously published protocols for phage panning in a cell culture setting (84). The panning method was adapted to select phages based on their ability to translocate into the target cell line, as staphylococcal osteomyelitis is associated with intracellular persisters (12). Murine pre-osteoblasts (MC3T3-E1) were seeded in a 12-well plate (4 × 10^5^ cells/well) and grown for 24 hours to 90% confluency. Cells were washed once with culture medium without FBS supplementation and incubated for 2 hours to clear cell surface receptors. Cells were incubated for 1 hour with 10^10^ PFU/mL phages from the Ph.D.-12 Phage Display Peptide Library in PBS^+^ (137 mM NaCl, 2.7 mM KCl, 10 mM Na_2_HPO_4_, 1.8 mM K_2_HPO_4_, 0.5 mM CaCl_2_, 10 mM MgCl_2_, pH = 7.4), supplemented with 0.1% bovine serum albumin (BSA) (Sigma-Aldrich), 0.1 mM chloroquine (Sigma-Aldrich), and 1× Protease Inhibitor (EDTA-free; Roche, Basel, Switzerland). Cells were washed five times for 5 minutes with PBS^+^ to remove unbound phage particles. Surface-bound phages were released by two acid washes (0.1 M glycine HCl, 0.9% [wt/vol] NaCl, pH = 2.2). Cells were lysed with 30 mM Tris-HCl (pH = 8) on ice, followed by one freeze-thaw cycle at −20°C. The phage-containing lysate was mixed with propagation strain *E. coli* K12 ER2738 (provided with the kit) at the early-log phase, plated on LB agar supplemented with 15 µg/mL tetracycline and incubated at 37°C for 20 hours. Phages were washed off the plates with LB medium and host cells were removed from the solution by centrifugation (3,000 × *g*, 10 minutes, 4°C). Phages were precipitated from supernatant by the addition of precipitation solution (2.5 M NaCl, 20% PEG-8000) at a ratio of 1:4 and incubated at 4°C for 20 hours. Precipitated phages were pelleted by centrifugation (11,000 × *g*, 30 minutes, 4°C) and resuspended in DPBS. Residual bacteria were heat-inactivated at 65°C for 15 minutes and insoluble debris was removed by centrifugation (16,000 × *g*, 10 minutes, 4°C). Phages were aliquoted and stored at −80°C. Phage stocks were titrated in *E. coli* K12 ER2738 LB soft-agar overlays on LB agar plates supplemented with 0.2 mM IPTG and 0.1 mM X-Gal (BioChemica, Billingham, UK) for blue-white screening.

### Illumina sequencing of phage pool displaying putative cell-penetrating homing peptides

To gain deeper insight into the peptides present in the phage pool, deep sequencing of selected phage pools was performed after one round of panning as proposed by t’Hoen nd colleagues (61). The 36 bp inserts in the phage genomes encoding the surface-displayed 12-mer peptides were amplified by PCR using primers M13_PhD_Insert_F and M13_PhD_Insert_R (Table S4). Amplicons were purified with the GeneElute PCR Clean-up Kit (Sigma-Aldrich) and eluded in water. The concentration was determined by spectrophotometric measurements and purity was assessed by gel electrophoresis on a 10% agarose gel (75 V, 90 minutes, 50–60 ng loaded). Amplicons were sequenced using Illumina HiSeq Amplicon sequencing (6,000,000 reads, 2 × 151 bp; GATC Biotech, Konstanz, Germany), and data were analyzed using the CLC Genomics Workbench (V9.5.4; Qiagen, Hilden, Germany) and Microsoft Excel (Microsoft, Redmond, USA). Several web-based tools were used to assess the obtained peptide sequences. The SAROTUP suite of online tools was used to identify possible target-unrelated peptides by comparing them to a database of peptides obtained in other panning experiments, and to known polystyrene-binding motifs or target-unrelated peptides (85, 86). The list of candidates obtained from panning on the target cell MC3T3-E1 was compared to a panning performed with the same library in primary murine heart endothelial cells (data not shown), and redundant peptides were excluded as unspecific. The remaining peptides were analyzed using the NCBI BlastP suite (NCBI, Rockville Pike, USA) to identify similarities of interest to proteins that naturally occur in the targeted niche.

### Cloning of EGFP_CPHP and PGH_CPHP constructs

All enzymes listed in Table S3 were constructed using standard molecular cloning techniques (87). Primers listed in Table S4 were purchased from Microsynth AG (Balgach, Switzerland). Sequences were verified by colony PCR and subsequent commercial Sanger sequencing (GATC Biotech).

#### H_eGFP_CPHP constructs

Ten CPHP candidates with putative specificity for pre-osteoblasts were genetically fused to the fluorescent protein eGFP via a flexible glycine- and serine-containing linker (L = GGSGGGSGG). GeneArt Strings (Thermo Fisher Scientific) of the structure *XhoI*_eGFP_*SacI*_L_CPHP_*BamHI* were digested using restriction enzymes XhoI and BamHI-HF (NEB). Digested strings were ligated into expression vector pET302_NT/HIS (Invitrogen, Waltham, USA), transformed into *E. coli* BL21-Gold(DE3), and plated on LB agar supplemented with appropriate antibiotics.

#### LST_CPHP constructs

To assess the intracellular activity of a PGH internalized into eukaryotic cells by selected CPHPs, the region coding for LST was amplified from plasmid pET302_LST, and 10 CPHP candidates were introduced through a specific reverse primer for each construct. The resulting amplicons of the structure *NdeI*_LST_*SacI*_CPHP_*BamHI* and the selection vector pET302_mCherry were digested. After ligation and transformation, clones were screened based on their color (white: replacement of mCherry in multiple cloning site (MCS) with insert of choice).

#### M23_CPHP and CHAPGH15_CPHP constructs

Constructs used for *in vivo* experiments were created using GeneArt Strings of the structure *NdeI*_PGH_*SacI*_CPHP_*BamHI*. Cloning was executed as described for LST_CPHPs above.

### Verification of cell-penetrating characteristics of CPHP candidates by fluorescence microscopy

Putative CPHPs were fused to eGFP to qualitatively assess their ability to penetrate and internalize into eukaryotic cells. Cell lines to be tested (MC3T3-E1, Caco-2) were seeded in 8-well µ-Slides (ibidi GmbH, Martinsried, Germany) at a concentration of 1–5 × 10^5^ cells/well, so that they reach 70–80% confluency the next day. Cells were washed once with cell culture medium without FBS and incubated for 1 hour. eGFP_CPHP constructs were diluted to 5 µM in culture medium, supplemented with the nucleic acid stain Hoechst 33342 (7.5 µg/mL; Thermo Fisher Scientific), and incubated with the cells for 1 hour. Cells were washed five times with cell culture medium and the membrane was stained by the addition of FM4-64 in culture medium (8 µM; Thermo Fisher Scientific). Cells were imaged on a Leica TCS SPE confocal microscope equipped with a HCX PL FLUOTAR 100.0 × 1.30 OIL objective (Leica Microsystems GmbH, Wetzlar, Germany). Excitation values for eGFP, Hoechst 33342, and FM4-64 were set at λ = 488, λ = 405, and λ = 532, respectively. All images were analyzed using the same methodology in the Leica Application Suite X (V2.0.0; Leica).

### Assessment of intracellular activity of a PGH cargo molecule

Intracellular killing assays were performed essentially as previously described (44). LST_CPHP constructs were expressed and purified, and *in vitro* activity was determined in a time-kill assay as described above. MC3T3-E1 cells were seeded in a 24-well plate (1 × 10^5^ cells/well) to reach 90% confluency the next day. Cells were washed once with culture medium and infected with *S. aureus* Cowan I at an MOI of 5. To enhance bacterial settling, the plate was subjected to centrifugation (2,000 × *g* for 5 minutes). The plate was incubated for 1 hour to allow for infection. After two wash steps, eukaryotic cells were incubated for 1 hour in a culture medium supplemented with flucloxacillin (1 mg/mL, Sigma-Aldrich) to select for intracellular bacteria. Eukaryotic cells were treated for 4 hours with 2 µM LST_CPHP. After three washes with DPBS, cells were detached with Trypsin and lysed with 0.1% Triton X-100 (both from Thermo Fisher Scientific). To ensure complete lysis, each well was mixed 40 times using a standard micropipette. DPBS was added to the lysate to a final volume of 1 mL, and 10-fold dilutions were spotted onto LB agar plates in triplicates. Plates were incubated overnight at 37°C and colony counts were determined the next day.

### Chemical activation of DTBTA-Eu^3+^ and labeling of LST and LST_CPHP variants

To assess the biodistribution of CPHP candidates, endotoxin-free enzymes were labeled with DTBTA-Eu^3+^ as described previously (44). DTBTA-Eu^3+^ is a fluorescent marker that can be measured using time-resolved fluorometry to eliminate background fluorescence in complex matrices (88). The active luminescent label {2,2′,2′′,2′′′-{[4′-{(4,6-dichloro-1,3,5-triazin-2-yl)aminobiphenyl-4-yl}−2,2′:6′,2′′-terpyridine-6,6′′-diyl]bis(methylene-nitrilo)}tetrakis(acetato)} europium(III) (DTBTA-Eu^3+^) was synthesized from the {2,2′,2′′,2′′′-{[4′-(aminobiphenyl-4-yl)−2,2′:6′,2′′-terpyridine-6,6′′-diyl]bis(methylenenitrilo)}tetrakis(acetato)} europium(III) chelate (ATBTA-Eu^3+^; TCI, Tokyo, Japan) essentially as described previously (88): 2 mg ATBTA-Eu^3+^ were dissolved in 60 µL 0.1 M NaAc (pH = 4.9). Twenty-five microliters of 90 mM cyanuric chloride in acetone was added to the label and stirred for 30 minutes at room temperature. The mixture was precipitated in acetone. Precipitated label was washed twice with acetone and lyophilized. Label conversion was verified by electrospray ionization mass spectrometry (ESI-MS) (Functional Genomics Center Zurich, Switzerland). The sample was diluted in 20 mM NH_4_Ac (50% MeOH, pH = 4.5), infused through a fused silica capillary (ID, 75 μm, 1 µL/min; New Objective, Woburn, USA), and sprayed through a PicoTip emitter (ID30um; New Objective). Nano ESI-MS analysis was performed on a Synapt G2-Si instrument (spray voltage 2.5 kV, cone voltage 40 V, source temperature 80°C; Waters, Milford, USA). Data were recorded with the MassLynx 4.2 Software (Waters). Mass spectra were acquired in the positive-ion mode, scanning an *m*/*z* range from 50 to 3,000 Da. Scan duration and interscan delay were set at 1 and 0.1 seconds, respectively. CIEX-purified proteins were dialyzed into carbonate buffer (100 mM NaHCO_3_, 100 mM Na_2_CO_3_, 300 mM NaCl, pH = 9.2), concentrated to 2.5 mL on Vivaspin 6 spin columns (MWCO 10,000; Sartorius, Göttingen, Germany), and concentration was determined using the Pierce BCA Protein Assay KIT (Thermo Fisher Scientific). LST and LST_CPHP variants were labeled with DTBTA-Eu^3+^ at a molar ratio of 4:1 (label:protein), incubating for 2.5 hours at room temperature under constant motion. To remove protein complexes cross-linked by the labeling process and unbound label, samples were additionally purified by SEC as described above.

### Determination of cytotoxicity levels of PGH and PGH_CPHP constructs to be tested *in vivo*

Cytotoxicity of PGH and PGH_CPHP constructs was determined using the CyQuant LDH Cytotoxicity Assay Kit (Invitrogen) according to the provided protocols. MC3T3-E1 cells were seeded in a 96-well plate (2.5 × 10^3^ cells/well) in a culture medium supplemented with FBS and grown for 24 hours. Enzymes diluted to 2 µM in a culture medium without FBS were added to the cells and incubated for 4 hours. Cytotoxicity compared to an untreated control was determined in technical and biological triplicates.

### Biodistribution of LST_CPHP candidates in mice

C57BL/6 mice (female, 8–10 weeks of age; Janvier Laboratory, Le Genest-Saint-Isle, France) were injected with 100–150 µL of DTBTA-Eu^3+^-labeled LST and LST_CPHP fusion proteins in DPBS at a concentration of 10 mg/kg of body weight (*n* =3) into the tail vein. LST fused to the short peptide SDSSD was included as a reference in these experiments since this peptide had previously been shown to accumulate in bone-formation surfaces in mice (59). Mice were euthanized 12 hours post-injection. Bones (femur, tibia, and fibula), kidneys, liver, heart, spleen, lung, and muscle (biceps femoris and quadriceps femoris) were collected, diluted in DPBS (kidney and liver 1:1; heart, lung, and muscles 1:2; spleen 1:3; bones 1:4) and homogenized. To quantify recovered signals, standard curves using known protein concentrations were prepared for each labeled protein in homogenized tissues of interest recovered from a control mouse, which had been injected with DBPS at the time of treatment. Since each DTBTA-^Eu3+^ labeling reaction may result in a different average number of Eu^3+^ labels per protein molecule, and consequently, identical concentrations of different labeled protein batches may yield different fluorescence signals, such standard curves were created for each labeled protein batch used in the biodistribution study to allow for accurate determination of protein concentrations from harvested tissue samples. Standards and treated tissue samples were measured on a Tecan plate reader (Tecan Infinite M1000; Tecan, Männedorf, Switzerland) using time-resolved fluorescence (excitation: 348 nm, emission: 619 nm, lag time: 275 µs, integration time: 500 µs). Measurements were fitted to standard curves and the amount of protein per tissue weight was calculated (*n* ≥ 3).

### Efficacy study in a murine deep wound subcutaneous infection model

A deep wound subcutaneous infection mouse model leading to dissemination to the bone was established based on the methodology of a previously published murine abscess model (89, 90). *S. aureus* Cowan I was grown to log phase, and 10^8^ CFU in 50 µL DPBS were mixed 1:1 with Cytodex beads (Sigma-Aldrich). Deep tissue infection was carried out in C57BL/6 mice (female, 8–10 weeks of age; Janvier Laboratory) by injecting 100 µL of the *S. aureus*-beads mixture subcutaneously in both hind legs. At 48 hours post-infection, mice were systemically treated via tail vein injection with 100 µL of PBS (untreated control) or PBS containing 75 µM of parental PGH, PGH_HGDWT, or PGH_SDSSD, and euthanized after a 14- to 16-hour treatment period. Mice femur, tibia, and fibula from both hind legs were harvested, homogenized, and serially diluted to enumerate bacterial CFUs on blood agar plates. CFU counts were normalized to the weight of the homogenized bone sample (gram tissue), yielding total CFUs per bone. CFU counts from both hind legs were averaged and used as biological replicate (*n* = 5–6).

### Statistical analysis

All statistical analyses shown were performed in GraphPad Prism (V9.2.0, GraphPad Software, San Diego, USA). Data obtained in the intracellular killing assay and time-kill assays were log transformed and analyzed using a Kruskal-Wallis test, followed by Dunn’s multiple comparison. Analysis of the biodistribution experiments was performed using an ordinary one-way ANOVA and subsequent multiple comparison with Dunnett’s correction, to compare the CPHP-labeled constructs to the unlabeled LST control. Data obtained in the efficacy study were analyzed using a Kruskal-Wallis test, followed by Dunn’s multiple comparison to compare the groups of parental PGH and PGH_CPHP variants to the PBS control. *P*-values < 0.05 were deemed significant, and *P*-values are represented in the figures as follows: ns, *P* > 0.05; *, *P* ≤ 0.05; **, *P* ≤ 0.01; ***, *P* ≤ 0.001; and ****, *P* < 0.0001.

## Data Availability

Data supporting the findings of this study are available within the article and its supplemental material files or from the corresponding author upon request.

## SUPPLEMENTAL MATERIAL

Figure S1 mbio.01830-23-s0001.pdf Verification of cell-penetrating characteristics of CPHP candidates.

Figure S2 mbio.01830-23-s0002.pdf Comparison of the activity of parental LST and LST fused to CPHP candidates.

Figure S3 mbio.01830-23-s0003.pdf Conversion of ATBTA-Eu^3+^ to DTBTA-Eu^3+^ was verified by ESI-MS.

Figure S4 mbio.01830-23-s0004.pdf Purity and cytotoxicity of PGHs produced for the efficacy study in a murine deep wound subcutaneous infection model.

Figure S5 mbio.01830-23-s0005.pdf Staphylolytic activity of parental and CPHP-fused PGHs used in an efficacy study in a murine deep wound subcutaneous infection model.

Table S1 mbio.01830-23-s0006.pdf Bacterial strains used in this study for functional assays (*S. aureus*) and expression of proteins (*E. coli*)

Table S2 mbio.01830-23-s0007.pdf Peptidoglycan hydrolases screened for high activity in murine serum.

Table S3 mbio.01830-23-s0008.pdf Protein constructs created and presented in this work.

Table S4 mbio.01830-23-s0009.pdf PCR primers used in this study.

ASM does not own the copyrights to Supplemental Material that may be linked to, or accessed through, an article. The authors have granted ASM a non-exclusive, world-wide license to publish the Supplemental Material files. Please contact the corresponding author directly for reuse.
