# Supplementary material for: Systemic application of bone-targeting peptidoglycan hydrolases as a novel treatment approach for staphylococcal bone infection
Source: mBio. 2023 Sep 28;14(5):e01830-23. doi: 10.1128/mbio.01830-23 (PMC10653945; doi:10.1128/mbio.01830-23)
Supplement: Figure S1 — Verification of cell-penetrating characteristics of CPHP candidates. [file mbio.01830-23-s0001.pdf]

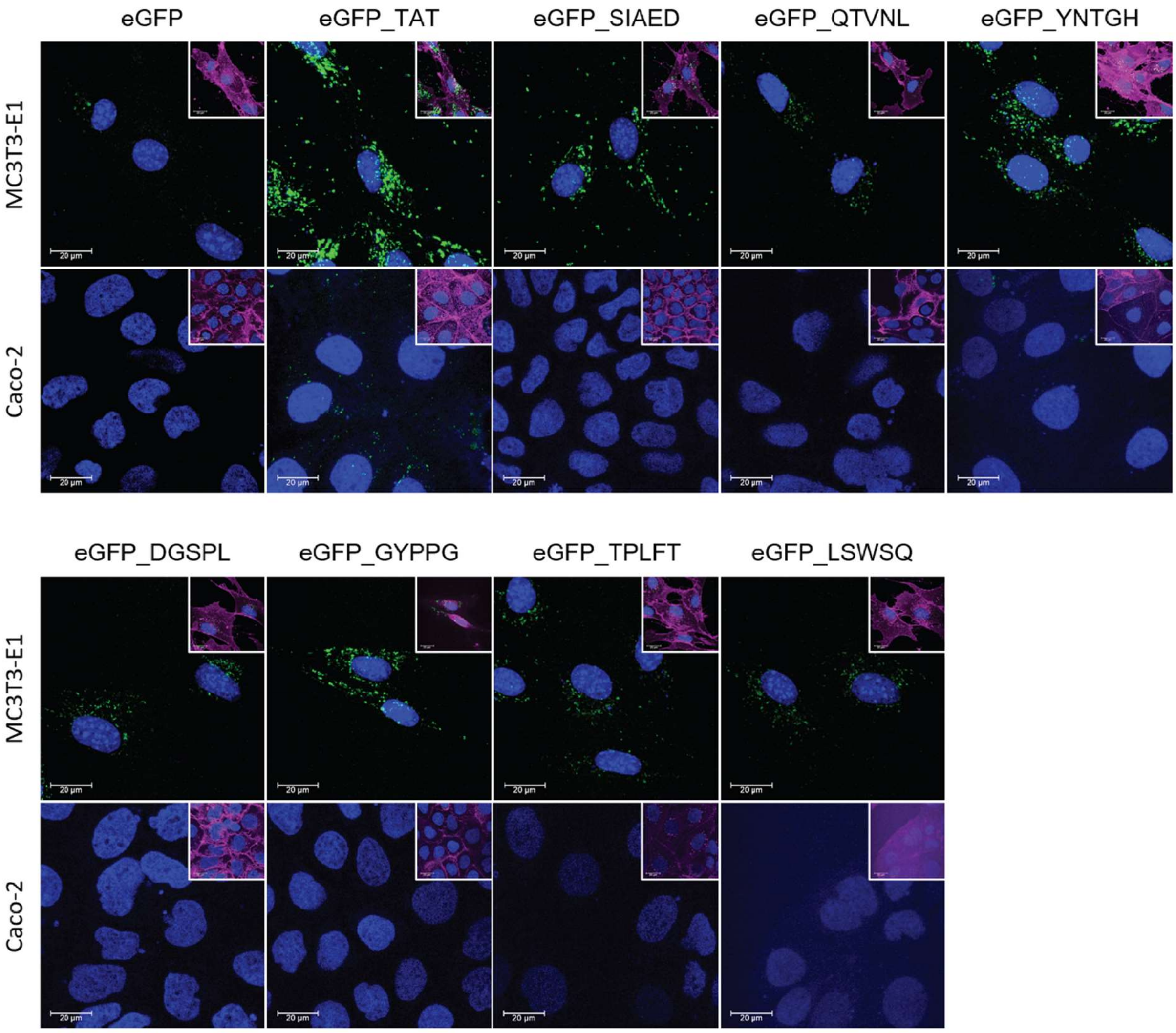

Supplementary Figure S1: Verification of cell-penetrating characteristics of CPHP candidates. Uptake of CPHP candidates identified by phage display on a murine pre-osteoblast cell line (MC3T3-E1) was analysed in the target cell line MC3T3-E1 (top rows) and a non-target cell line (Caco-2, bottom rows). Cells were treated for 60 minutes with 5 µM eGFP_CPHP and the controls eGFP and eGFP_TAT (general cell-penetrating peptide), washed with DPBS to remove extracellular signal and cells were stained with FM4-64 (membrane, magenta) and Hoechst 33342 (DNA, blue). Cell-line specific uptake of eGFP_CPHP was assessed by CLSM. Here, the seven CPHP candidates that were not chosen for subsequent *in vivo* experiments are shown. Please note that images for controls (eGFP and eGFP_TAT) are identical to those presented in Figure 3B. An overlay including the stained membrane is shown separately to the overlay of the nucleic acid and eGFP signal for ease of visualization.
