# Supplementary material for: Systemic application of bone-targeting peptidoglycan hydrolases as a novel treatment approach for staphylococcal bone infection
Source: mBio. 2023 Sep 28;14(5):e01830-23. doi: 10.1128/mbio.01830-23 (PMC10653945; doi:10.1128/mbio.01830-23)
Supplement: Figure S2 — Comparison of the activity of parental LST and LST fused to CPHP candidates. [file mbio.01830-23-s0002.pdf]

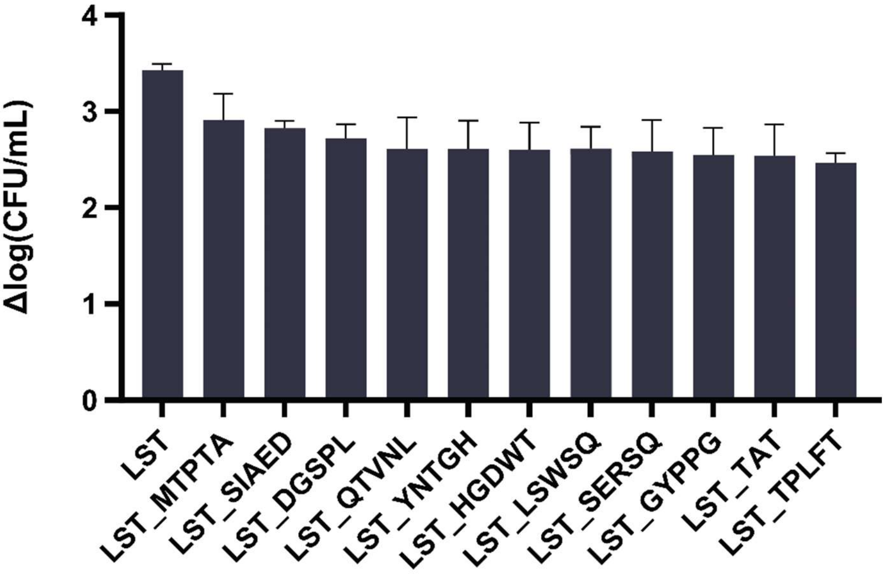

Supplementary Figure S2: Comparison of the activity of parental LST and LST fused to CPHP candidates. *S. aureus* Cowan I (10^6 CFU/mL) in cell culture medium (AMEM) was treated with 20 nM enzyme for 120 minutes. Surviving bacteria were enumerated by plating and the average log(CFU/mL) reduction compared to the untreated control was determined (± SEM) from biological triplicates. No significant differences were observed for the modified constructs as compared to the unmodified LST.
