# Supplementary material for: Systemic application of bone-targeting peptidoglycan hydrolases as a novel treatment approach for staphylococcal bone infection
Source: mBio. 2023 Sep 28;14(5):e01830-23. doi: 10.1128/mbio.01830-23 (PMC10653945; doi:10.1128/mbio.01830-23)
Supplement: Figure S3 — Conversion of ATBTA-Eu3+ to DTBTA-Eu3+ was verified by ESI-MS. [file mbio.01830-23-s0003.pdf]

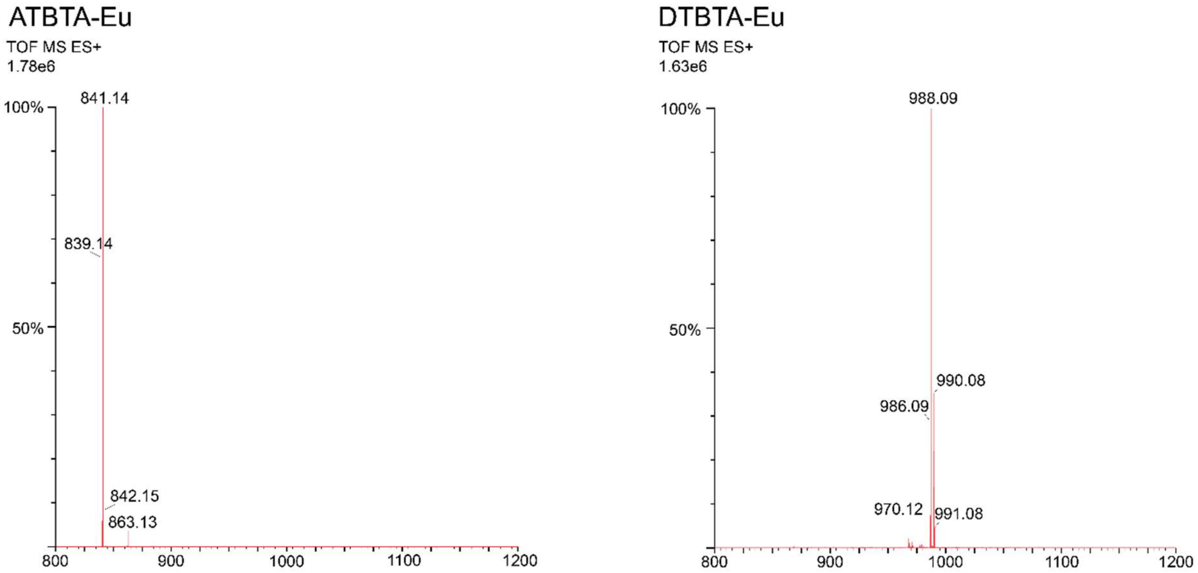

Supplementary Figure S3: Conversion of ATBTA-Eu^{3+} to DTBTA-Eu^{3+} was verified by ESI-MS. ATBTA-Eu^{3+} (left, 861.5 (M − H$^+$)$^-$, 839.5 (M − Na$^+$)$^-$) was chemically converted to the activated DTBTA-Eu^{3+} (right, 1008.3 (M − H$^+$)$^-$, 986.3 (M − Na$^+$)$^-$, 968.3 (M − H_2O)$^-$), which was used to label LST and LST_CPHPs for biodistribution experiments. The mass spectrum on the right confirms complete conversion of the label and was chosen as a representative sample for each batch of activated DTBTA-Eu^{3+} produced for this study.
