# Supplementary material for: Systemic application of bone-targeting peptidoglycan hydrolases as a novel treatment approach for staphylococcal bone infection
Source: mBio. 2023 Sep 28;14(5):e01830-23. doi: 10.1128/mbio.01830-23 (PMC10653945; doi:10.1128/mbio.01830-23)
Supplement: Figure S4 — Purity and cytotoxicity of PGHs produced for the efficacy study in a murine deep wound subcutaneous infection model. [file mbio.01830-23-s0004.pdf]

A

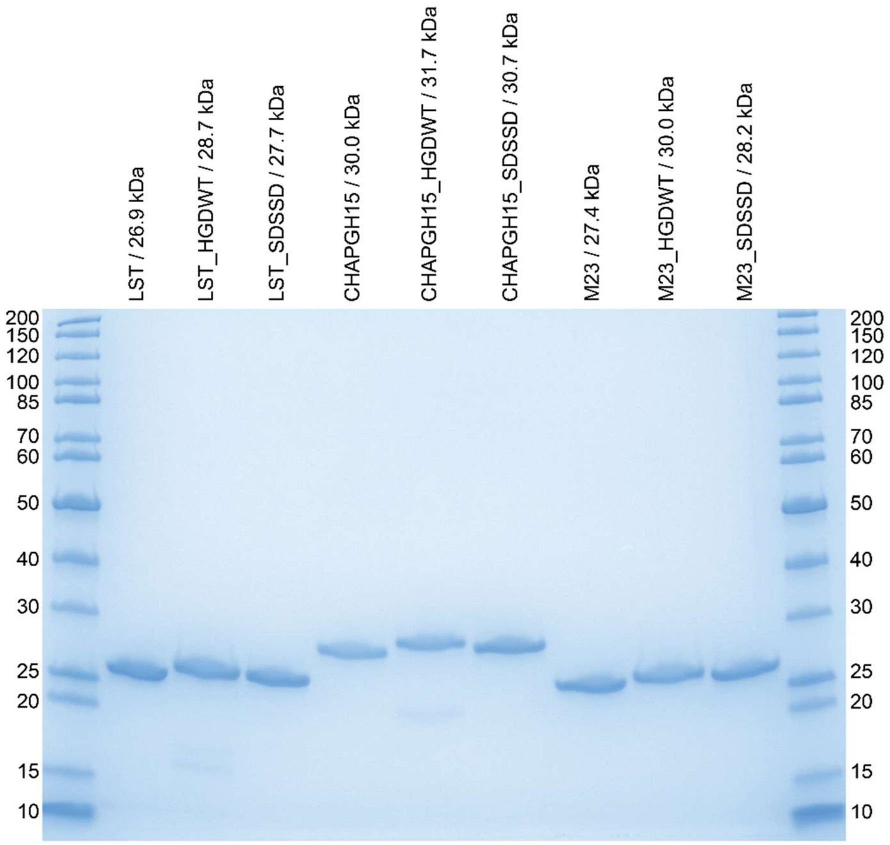

B

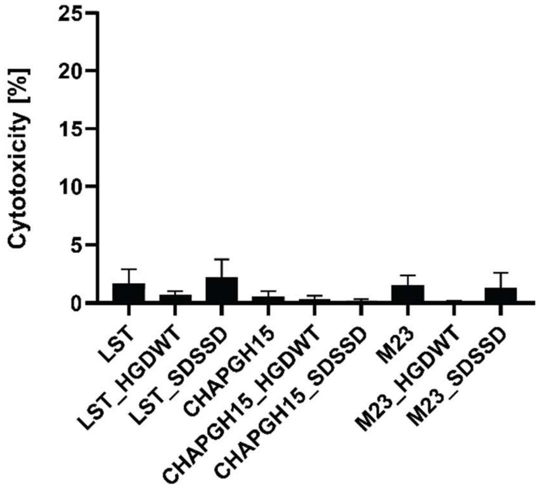

Supplementary Figure S4: Purity and cytotoxicity of PGHs produced for the efficacy study in a murine deep wound subcutaneous infection model. (A) Purity of proteins expressed in an endotoxin-free manner and purified by cation exchange and size exclusion chromatography was assessed by SDS-PAGE. 2 µg of each enzyme was loaded. The PageRuler^{TM} unstained 200 kDa ladder (Thermo Fisher Scientific, Waltham, US) was used for size reference. **(B)** Cytotoxicity of enzymes in eukaryotic cells was determined by treating MC3T3-E1 cells with 2 µM enzyme for 4 hours and measuring LDH levels in the supernatant with the CyQuant™ LDH cytotoxicity assay kit (Invitrogen). Percentage cytotoxicity was calculated by division of the signals of the samples by the signal obtained from complete cell lysis with a detergent, and multiplication by 100%. Plotted values represent the average cytotoxicity (± SEM) obtained from biological triplicates.
