# Supplementary material for: Systemic application of bone-targeting peptidoglycan hydrolases as a novel treatment approach for staphylococcal bone infection
Source: mBio. 2023 Sep 28;14(5):e01830-23. doi: 10.1128/mbio.01830-23 (PMC10653945; doi:10.1128/mbio.01830-23)
Supplement: Figure S5 — Staphylolytic activity of parental and CPHP-fused PGHs used in an efficacy study in a murine deep wound subcutaneous infection model. [file mbio.01830-23-s0005.pdf]

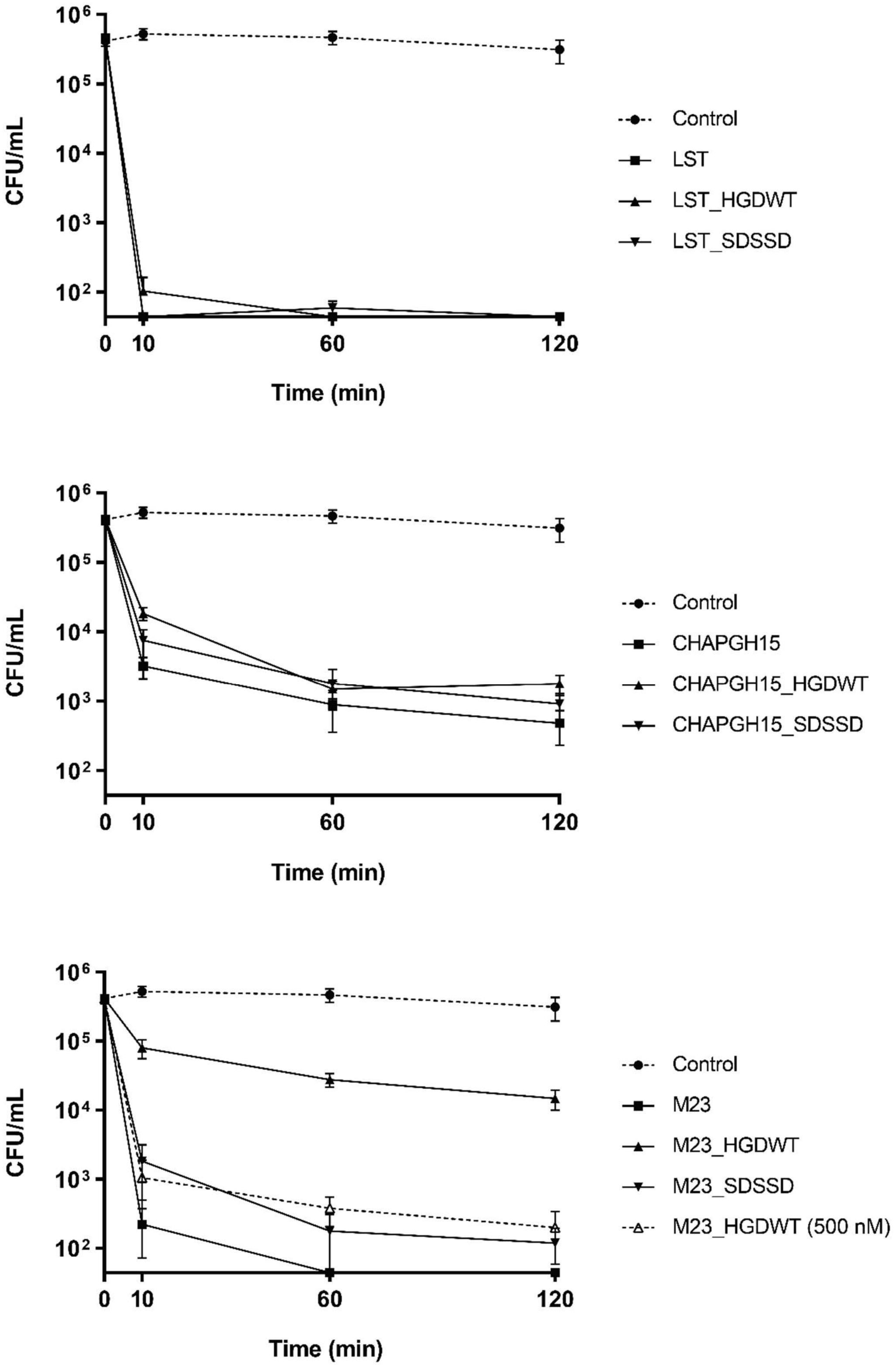

Supplementary Figure S5: Staphylolytic activity of parental and CPHP-fused PGHs used in an efficacy study in a murine deep wound subcutaneous infection model. *S. aureus* Cowan I (10^6 CFU/mL) was treated with 20 nM enzyme in murine serum. Bacterial counts were determined after 10, 60, and 120 minutes by plating. Plotted are the mean CFU/mL (± SEM) determined at each time point in biological triplicates. The Y-axis was cut at the detection limit (44 CFU/mL).
