# Supplementary material for: Systemic application of bone-targeting peptidoglycan hydrolases as a novel treatment approach for staphylococcal bone infection
Source: mBio. 2023 Sep 28;14(5):e01830-23. doi: 10.1128/mbio.01830-23 (PMC10653945; doi:10.1128/mbio.01830-23)
Supplement: Table S1 — Bacterial strains used in this study for functional assays (S. aureus) and expression of proteins (E. coli) [file mbio.01830-23-s0006.pdf]

Supplementary Table S1: Bacterial strains used in this study for functional assays (*S. aureus*) and expression of proteins (*E. coli*).

Species	Strain	Comments	Source
S. aureus	Newman	Clinical isolate (MSSA)	ATCC 25904 (1)
S. aureus	Cowan I	Clinical isolate (MSSA)	ATCC 12598 (2)
S. aureus	USA300 JE2	Clinical isolate (MRSA)	NARSA NR-46543 (3)
E. coli	BL21-Gold(DE3)	Tet^R	Agilent Technologies, Santa Clara, US
E. coli	SURE	Tet^R, Kan^R	Stratagene, La Jolla, US
E. coli	XL1-Blue MRF’	Tet^R	Stratagene, La Jolla, US
E. coli	ClearColi® BL21(DE3)	LTA-deficient strain for endotoxin-free protein expression	Lucigen, Middleton, US

Abbreviations: ATCC, American Type Culture Collection; Kan^R, Kanamycin resistance; LTA. lipoteichoic acid; MRSA, methicillin-resistant *Staphylococcus aureus*; MSSA, methicillin-sensitive *Staphylococcus aureus*; NARSA, Network on Antimicrobial Resistance in *Staphylococcus aureus*; Tet^R, tetracycline resistance.

1. Duthie ES, Lorenz LL. 1952. Staphylococcal coagulase; mode of action and antigenicity. J Gen Microbiol 6:95-107.
2. Bohacek J, Kocur M, Martinec T. 1971. Deoxyribonucleic acid base composition of serotype strains of *Staphylococcus aureus*. J Gen Microbiol 68:109-13.
3. Fey PD, Endres JL, Yajjala VK, Widhelm TJ, Boissy RJ, Bose JL, Bayles KW. 2013. A genetic resource for rapid and comprehensive phenotype screening of nonessential *Staphylococcus aureus* genes. mBio 4:e00537-12.
