# Supplementary material for: Systemic application of bone-targeting peptidoglycan hydrolases as a novel treatment approach for staphylococcal bone infection
Source: mBio. 2023 Sep 28;14(5):e01830-23. doi: 10.1128/mbio.01830-23 (PMC10653945; doi:10.1128/mbio.01830-23)
Supplement: Table S2 — Peptidoglycan hydrolases screened for high activity in murine serum. [file mbio.01830-23-s0007.pdf]

Supplementary Table S2: Peptidoglycan hydrolases screened for high activity in murine serum. All constructs are part of the strain collection of the Laboratory of Food Microbiology (ETH, Zurich).

	ID	Name	Plasmid[1]	*E. coli* expression strain
Gly-Gly Endopeptidase	**PGH 1**	H_(M23LST)2_LST	pQE30	SURE
	**PGH 2**	M23LST(L)_SH3b2638	pET9a	BL21-Gold(DE3)
	**PGH 3**	H_(LST)2	pQE30	SURE
	**PGH 4**	H_LST_M23LST	pQE30	SURE
	**PGH 5**	H_TEV_(M23LST)2_SH3bLST	pQE30	SURE
	**PGH 6**	(M23LST)2_SH3b2638	pET302[1]	BL21-Gold(DE3)
	**PGH 7**	(M23LST)2_SH3bAle1	pET302	BL21-Gold(DE3)
Gly-D-Ala Endopeptidase	**PGH 8**	CHAPGH15_SH3bAle1	pET302	BL21-Gold(DE3)
	**PGH 9**	CHAPTw(L)_SH3b2638	pET302	BL21-Gold(DE3)
	**PGH 10**	H_CHAP-1_CBD-1	pET302	BL21-Gold(DE3)
	**PGH 11**	H_TEV_CHAPK_SH3bLST	pQE30	XL1-Blue MRF'
	**PGH 12**	CHAPK_SH3bLST_H	pET21a	BL21-Gold(DE3)
	**PGH 13**	CHAPGH15_SH3b2638	pET302	BL21-Gold(DE3)
	**PGH 14**	CHAPSEP1(L)_SH3b2638	pET302	BL21-Gold(DE3)
	**PGH 15**	(CHAPGH15)2_SH3bAle1	pET302	BL21-Gold(DE3)
	**PGH 16**	H_TEV_(CHAPK)2_SH3bLST	pQE30	SURE
	**PGH 17**	(CHAPGH15(L))2_SH3b2638	pET302	BL21-Gold(DE3)
Double EAD mixed specificity	**PGH 18**	CHAPTw_M23LST(L)_SH3b2638	pET302	BL21-Gold(DE3)
	**PGH 19**	CHAPK_AmiK_SH3bLST_H	pET21a	BL21-Gold(DE3)
	**PGH 20**	LysK_H	pET21a	BL21-Gold(DE3)
	**PGH 21**	M23LST(L)_Ami2638_SH3b2638	pET200	BL21-Gold(DE3)
	**PGH 22**	M23LST(L)_Ami2638(L)_SH3b2638	pET200	BL21-Gold(DE3)
Triple EAD mixed specificity	**PGH 23**	LST_CHAPK_AmiK_H	pET21a	BL21-Gold(DE3)
	**PGH 24**	LST_CHAPH5_AmiH5(L)_H	pET21a	BL21-Gold(DE3)
	**PGH 25**	LysK_LST_H	pET21a	BL21-Gold(DE3)
	**PGH 26**	PepλSA2_CHAPK_LST_H	pET21a	BL21-Gold(DE3)
	**PGH 27**	CHAP11_Ami11_LST_H	pET21a	BL21-Gold(DE3)
	**PGH 28**	CHAPH5_AmiH5_LST_H	pET21a	BL21-Gold(DE3)

[1] Resistance encoded on plasmid: ampicillin on pQE30, pET302 and pET21a; kanamycin on pET9a and pET21a.

Abbreviations: EAD, enzymatically active domain; H, 6x histidine tag; PGH, peptidoglycan hydrolase.
