# Supplementary material for: Systemic application of bone-targeting peptidoglycan hydrolases as a novel treatment approach for staphylococcal bone infection
Source: mBio. 2023 Sep 28;14(5):e01830-23. doi: 10.1128/mbio.01830-23 (PMC10653945; doi:10.1128/mbio.01830-23)
Supplement: Table S3 — Protein constructs created and presented in this work. [file mbio.01830-23-s0008.pdf]

Supplementary Table S3: Protein constructs created and presented in this work. All constructs were cloned into the expression vector pET302.

ID	Name	Expression strain[1]	Origin[2]	Application[3]
**eGFP**	H_eGFP	A	1	FM
**eGFP_SERSQ**	H_eGFP_L_SERSQYIPSTRT	A	1	FM
**eGFP_QTVNL**	H_eGFP_L_QTVNLLSDVRRS	A	1	FM
**eGFP_DGSPL**	H_eGFP_L_DGSPLPQKIHPS	A	1	FM
**eGFP_YNTGH**	H_eGFP_L_YNTGHTPVKLHT	A	1	FM
**eGFP_HGDWT**	H_eGFP_L_HGDWTKRWSFLA	A	1	FM
**eGFP_GYPPG**	H_eGFP_L_GYPPGAGHANTL	A	1	FM
**eGFP_TPLFT**	H_eGFP_L_TPLFTQEATGAN	A	1	FM
**eGFP_MTPTA**	H_eGFP_L_MTPTANTCVRCN	A	1	FM
**eGFP_LSWSQ**	H_eGFP_L_LSWSQTQGVSST	A	1	FM
**eGFP_SIAED**	H_eGFP_L_SIAEDQTISTPK	A	1	FM
**eGFP_TAT**	H_eGFP_L_TAT	A	1	FM
**LST**	LST	A/B	2	AA/BD/ES
**LST_SERSQ**	LST_SERSQYIPSTRT	A/B	1	AA/BD
**LST_QTVNL**	LST_QTVNLLSDVRRS	A	1	AA
**LST_DGSPL**	LST_DGSPLPQKIHPS	A	1	AA
**LST_YNTGH**	LST_YNTGHTPVKLHT	A	1	AA
**LST_HGDWT**	LST_HGDWTKRWSFLA	A/B	1	AA/BD/ES
**LST_GYPPG**	LST_GYPPGAGHANTL	A	1	AA
**LST_TPLFT**	LST_TPLFTQEATGAN	A	1	AA
**LST_MTPTA**	LST_MTPTANTCVRCN	A/B	1	AA/BD
**LST_LSWSQ**	LST_LSWSQTQGVSST	A	1	AA
**LST_SIAED**	LST_SIAEDQTISTPK	A	1	AA
**LST_SDSSD**	LST_SDSSD	A/B	1	AA/BD/ES
**LST_DSS6**	LST_DSS6	A	1	AA
**LST_TAT**	LST_TAT	A/B	2	AA
**M23**	M23LST(L)_SH3b2638	A/B	2	AA/ES
**M23_HGDWT**	M23LST(L)_SH3b2638_HGDWT	B	1	AA/ES
**M23_SDSSD**	M23LST(L)_SH3b2638_SDSSD	B	1	AA/ES
**CHAPGH15**	CHAPGH15_SH3bALE1	B	2	AA/ES
**CHAPGH15_HGDWT**	CHAPGH15_SH3bALE1_HGDWT	B	1	AA/ES
**CHAPGH15_SDSSD**	CHAPGH15_SH3bALE1_SDSSD	B	1	AA/ES

[1] Expression strains: A, *E. coli* BL21-Gold(DE3); B, *E. coli* ClearColi® BL21(DE3).

[2] Origin of construct: 1, this study; 2, strain collection of Laboratory of Food Microbiology, ETH Zurich, Zurich.

[3] Application: FM, fluorescence microscopy; AA, activity assays; BD, biodistribution; ES, efficacy study.
