# Supplementary material for: Systemic application of bone-targeting peptidoglycan hydrolases as a novel treatment approach for staphylococcal bone infection
Source: mBio. 2023 Sep 28;14(5):e01830-23. doi: 10.1128/mbio.01830-23 (PMC10653945; doi:10.1128/mbio.01830-23)
Supplement: Table S4 — PCR primers used in this study. [file mbio.01830-23-s0009.pdf]

Supplementary Table S4: PCR primers used in this study.

ID	Sequence (5' - 3')	Application
M13_PhD_Insert_F	TTCGCAATTCCTTTAGTGGT	Amplification of surface-displayed 12-mer peptide region in phage M13 genome of phage display-selected pools (Illumina sequencing)
M13_PhD_Insert_R	AAGTTTTGTCGTCTTTCCAG	
T7 seq	GAAATTAATACGACTCACTATAGGG	Amplification of MCS in pET302, sequencing primers (Sanger sequencing)
pET RP	CTAGTTATTGCTCAGCGG	
*NdeI*_LST_F	TTTCATATGGCAGCAACCCATGAACAT	Amplification of LST from pET302_LST and 5' incorporation of NdeI restriction site
LST_*SacI*_SERSQ_*BamHI*_R	GCCGGATCCTTAGGTACGTGTGCTCGGAATATACTGGCTACGTTCTGAGAGCTCTTTGATGGTGCCCCACAGAA	Amplification of LST from pET302_LST and 3' incorporation of CPHP and BamHI restriction site
LST_*SacI*_QTVNL_BamHI_R	GCCGGATCCTTAGCTACGACGAACATCACTCAGCAGATTAACGGTCTGGAGCTCTTTGATGGTGCCCCACAGAA	
LST_*SacI*_DGSPL_*BamHI*_R	GCCGGATCCTTAGCTCGGATGAATTTTCTGCGGCAGCGGACTGCCATCGAGCTCTTTGATGGTGCCCCACAGAA	
LST_*SacI*_YNTGH_*BamHI*_R	GCCGGATCCTTAGGTGTGCAGTTTAACCGGTGTATGACCTGTGTTATAGAGCTCTTTGATGGTGCCCCACAGAA	
LST_*SacI*_HGDWT_*BamHI*_R	GCCGGATCCTTATGCCAGAAACCAGCTACGTTTGGTCCAATCACCATGGAGCTCTTTGATGGTGCCCCACAGAA	
LST_*SacI*_GYPPG_*BamHI*_R	GCCGGATCCTTACAGGGTATTTGCATGACCTGCACCAGGCGGATAACCGAGCTCTTTGATGGTGCCCCACAGAA	
LST_*SacI*_TPLFT_*BamHI*_R	GCCGGATCCTTAATTTGCGCCTGTTGCTTCTTGGGTAAACAGCGGTGTGAGCTCTTTGATGGTGCCCCACAGAA	
LST_*SacI*_MTPTA_*BamHI*_R	GCCGGATCCTTAATTACAACGAACACAGGTATTTGCGGTCGGGGTCATGAGCTCTTTGATGGTGCCCCACAGAA	
LST_*SacI*_LSWSQ_*BamHI*_R	GCCGGATCCTTAGGTGCTGCTAACACCCTGGGTCTGTGACCAGCTCAGGAGCTCTTTGATGGTGCCCCACAGAA	
LST_*SacI*_SIAED_*BamHI*_R	GCCGGATCCTTATTTCGGGGTGCTAATGGTCTGATCTTCTGCAATTGAGAGCTCTTTGATGGTGCCCCACAGAA	
LST_*SacI*_SDSSD_*BamHI*_R	GCCGGATCCTTAATCACTGCTATCGCTGAGCTCTTTGATGGTGCCCCACAGAA	

Abbreviations: CPHP, cell-penetrating homing peptide; F, forward primer; MCS, multiple cloning site; R, reverse primer.
